# Eight characteristics of rigorous multilevel implementation research: a step-by-step guide

**DOI:** 10.1186/s13012-023-01302-2

**Published:** 2023-10-23

**Authors:** Rebecca Lengnick-Hall, Nathaniel J. Williams, Mark G. Ehrhart, Cathleen E. Willging, Alicia C. Bunger, Rinad S. Beidas, Gregory A. Aarons

**Affiliations:** 1 The Brown School, Washington University in St. Louis, St. Louis, MO USA; 2 School of Social Work, Boise State University, Boise, ID USA; 3 Department of Psychology, University of Central Florida, Orlando, FL USA; 4 Southwest Center, Pacific Institute for Research and Evaluation, Albuquerque, NM USA; 5 College of Social Work, The Ohio State University, Columbus, OH USA; 6 Medical Social Sciences, Feinberg School of Medicine, Northwestern University, Chicago, IL USA; 7 Department of Psychiatry, UC San Diego ACTRI Dissemination and Implementation Science Center, University of California-San Diego, La Jolla, San Diego, CA USA

**Keywords:** Multilevel, Research methods, Research reporting, Guideline, Research best practices

## Abstract

**Background:**

Although healthcare is delivered in inherently multilevel contexts, implementation science has no widely endorsed methodological standards defining the characteristics of rigorous, multilevel implementation research. We identify and describe eight characteristics of high-quality, multilevel implementation research to encourage discussion, spur debate, and guide decision-making around study design and methodological issues.

**Recommendations:**

Implementation researchers who conduct rigorous multilevel implementation research demonstrate the following eight characteristics. First, they map and operationalize the specific multilevel context for defined populations and settings. Second, they define and state the level of each construct under study. Third, they describe how constructs relate to each other within and across levels. Fourth, they specify the temporal scope of each phenomenon at each relevant level. Fifth, they align measurement choices and construction of analytic variables with the levels of theories selected (and hypotheses generated, if applicable). Sixth, they use a sampling strategy consistent with the selected theories or research objectives and sufficiently large and variable to examine relationships at requisite levels. Seventh, they align analytic approaches with the chosen theories (and hypotheses, if applicable), ensuring that they account for measurement dependencies and nested data structures. Eighth, they ensure inferences are made at the appropriate level. To guide implementation researchers and encourage debate, we present the rationale for each characteristic, actionable recommendations for operationalizing the characteristics in implementation research, a range of examples, and references to make the characteristics more usable. Our recommendations apply to all types of multilevel implementation study designs and approaches, including randomized trials, quantitative and qualitative observational studies, and mixed methods.

**Conclusion:**

These eight characteristics provide benchmarks for evaluating the quality and replicability of multilevel implementation research and promote a common language and reference points. This, in turn, facilitates knowledge generation across diverse multilevel settings and ensures that implementation research is consistent with (and appropriately leverages) what has already been learned in allied multilevel sciences. When a shared and integrated description of what constitutes rigor is defined and broadly communicated, implementation science is better positioned to innovate both methodologically and theoretically.

**Supplementary Information:**

The online version contains supplementary material available at 10.1186/s13012-023-01302-2.

Contributions to the literature

- Awareness of what constitutes rigorous multilevel implementation research is essential for theory generation and refinement across the diverse contexts in which implementation research is conducted.
- The methodological standards explained and recommended here are critical for planning, evaluating, and replicating multilevel implementation research.
- This manuscript articulates eight characteristics of rigorous, high-quality multilevel implementation research and provides prompts, topic-specific references, and implementation examples to help readers incorporate these ideas into their studies.

## Background

Rigorous implementation science requires transparent acknowledgment and skillful incorporation of the context within which implementation occurs. For implementation researchers, this requirement means addressing the inherently multilevel contexts within which healthcare is delivered. Patients who access healthcare are typically nested within one or more individual providers who deliver care (we use the term “providers” inclusively to encompass clinicians, practitioners, and others involved in health service delivery). Individual providers often work in one or more teams, clinics, or other subunits of organizations. Organizations, in turn, are typically embedded within one or more broader communities, networks, and systems. If the goal of implementation science is to improve patient and public health through “the study of methods to promote the systematic uptake of research findings and evidence-based practices (EBPs) into routine healthcare delivery” [1], we believe the field’s methods must rigorously address this complex, multilevel reality.

## When are multilevel methods necessary in implementation science?

Given the multilevel nature of healthcare and public health service delivery, we propose that implementation researchers should always *start* with the default assumption that their research design will need to address multilevel context and related methodological issues, *moving away* from this assumption only after confirming that all the methodological decisions made place the study design completely in “single-level” research territory. A design is “single level” when all phenomena and theoretical constructs of interest occur at the same level within the implementation context, all observations and measurements occur at that level, and there is neither theoretically nor empirically important nesting of research participants or dependence of observations (as might be caused, for example, by longitudinal measurement of providers working in the same unit). Although single-level conditions could be met in implementation studies, we propose it is extremely rare. We believe the burden is on implementation scientists (as developers, consumers, and evaluators of research) to ensure multilevel methodological issues are properly addressed in every implementation study.

## Challenges of multilevel research

Conducting methodologically rigorous multilevel studies is challenging. Such studies are often more complicated to design and execute than single-level studies [2]. Two (of many potential) examples of this complexity are difficulties associated with measuring implementation strategy and health intervention effects on outcomes at different levels and estimating their interaction effects across different levels [3]. As a result, conducting multilevel research tends to require a specific research skillset and a transdisciplinary approach [2, 4]. Here, we use Choi and Pak’s definition (p. 359): “Transdisciplinarity integrates the natural, social and health sciences in a humanities context, and in so doing transcends each of their traditional boundaries”[5].

The multilevel research literature is highly specialized and dispersed across different disciplinary journals, which hinders a researcher’s ability to access and synthesize existing guidance, especially for those who do not have multilevel research training [6]. This training includes firm grounding in foundational multilevel literature (e.g., Kozlowski & Klein’s seminal book [7]) and the focused study of key theories (e.g., psychological theories that explain multilevel organizational behavior), constructs (e.g., emergence, “shared unit” constructs), and methodological approaches (e.g., quantitative multilevel modeling).

Acknowledging and accounting for the multilevel structure in implementation contexts can also be laborious, resource intensive, and costly [2, 3, 8]. Practical challenges include getting appropriate expertise on the research team, recruiting and enrolling a large number of organizations or service systems (each of which has different gatekeepers with varying priorities/concerns), completing informed consent procedures with multiple levels of interconnected participants, and managing varying concerns about protecting participant confidentiality (e.g., collecting data that could identify participants but are considered standard demographic information such as employee age and number of years at the organization) [8]. Obstacles can arise when university ethics committees are unfamiliar with multilevel designs and have to make judgment calls about what constitutes coercion (e.g., staff feeling pressure to participate by their organizational leaders), how to operationalize informed consent in multilevel contexts, and who owns and houses the data [8].

We recognize that currently, the supply for multilevel expertise in implementation science is low, and the demand is high, especially given the field’s relatively untapped relationships with partners who have this expertise (e.g., faculty in business schools). As such, at this time, it is not reasonable to expect every implementation research team to include a multilevel research expert who has all of the aforementioned training. Therefore, we write this paper with the hope that it is a first step in exposing the implementation research community to key multilevel research topics and resources such that we can begin to build capacity for conducting and elevate the quality of existing, multilevel research across the field as a whole.

## Current literature

Researchers from several different disciplines have offered guidelines addressing multilevel research topics. Focusing on quantitative studies, González-Romá and Hernández [9] compiled an excellent list of multilevel topics, corresponding recommendations, and references. Topics include when and why multilevel methods are used, developing multilevel hypotheses, deciding between different quantitative analytic approaches (e.g., conventional multilevel modeling or multilevel structural equation modeling), and fitting a multilevel model [9]. As is evident in their table, each topic (1) covers content from its own separate set of references, (2) makes unique assumptions about the background knowledge readers need in order to follow the recommendations presented, and (3) is often field specific (e.g., management), a concern raised by Mathieu and Chen [4]. González-Romá and Hernández’s [9] table also highlights a dominant approach in the current set of multilevel research recommendations, that is, recommendations focused on quantitative multilevel modeling and specific topics therein [6, 10–12]. Other existing literature includes broad reflections on the state of multilevel research in the context of a specific field (i.e., absent detailed design guidance) [8] and discussions related to the design and evaluation of multilevel interventions (a subtopic within the multilevel research field) [13].

## The predicament of the implementation scientist interested in conducting multilevel research

Our eight characteristics draw from a realist ontological perspective, which holds that “entities exist independently of being perceived and independently of our theories about them” [14], as well as the multiple epistemological positions reflected within our authorship group and applied to projects depending on the research aims (e.g., post-positivism, social constructionism). We provide practical recommendations that are broadly applicable to all types of implementation research methodologies (i.e., quantitative, qualitative, and mixed methods). These recommendations are also relevant to any implementation research aim (e.g., implementing research-supported interventions, complex multilevel clinical practices, public health interventions, or policies) or study design (e.g., trials, observational studies) conducted at any level (or levels) of implementation contexts.

Again, recognizing that not every implementation researcher is, or can easily access, a multilevel research expert, we write this paper with these three goals in mind. First, to ease the reader’s burden of digesting a large body of specialized and divergent existing literature, we offer a cohesive set of research characteristics presented in a sequence that aligns with developing a research project (from research question formulation to evaluation). Second, to ease the burden of learning a new disciplinary language and reference points, we translate ideas from existing literature using constructs and practice examples familiar to an implementation research audience. Third, to be more inclusive of qualitative and mixed methods, we expand our focus beyond quantitative multilevel modeling. In sum, we echo Molina-Azorin and colleagues [2], with the intent of addressing the needs of the diverse implementation research community:

Our approach will be to see the ‘forest’ rather than some particular ‘trees.’ We examine the big picture, indicating the main elements of multilevel research. An exhaustive analysis of all the elements of multilevel research goes beyond the purpose of this methodological insight, but we provide key references in the literature that could be used…[with the hope that]…multilevel research brings us closer to the reality of [implementation] practice. pg. 2

## Road map for this paper

Our list of eight characteristics can be used to inform new research or enhance existing studies. We also hope that journal editors, peer reviewers, and funders will use this information when assessing the quality of multilevel implementation research. Each characteristic below is a continuation of the following sentence stem: “To conduct rigorous, high-quality multilevel implementation research…” In the text, we provide the rationale for each characteristic’s inclusion and recommendations for its operationalization when designing or evaluating research. The Additional Files 1–8 accompanying each characteristic illustrate how readers can apply it practically and concretely. Additional file documents feature prompts, practical considerations, checklists, visual aids, curated references, additional implementation research examples, notes about applicable glossary terms, and detailed guidance for navigating particular issues (e.g., creating a multilevel sampling plan). For readers interested in a holistic view of how our characteristics apply to a single study, we offer Additional File 9, which demonstrates the application of the characteristics in a mixed-methods, hybrid type III effectiveness-implementation trial called ASPIRE (for Adolescent and child Suicide Prevention in Routine clinical Encounters) [15]. The ASPIRE trial offers a unified, if imperfect, example of the characteristics because it incorporates (a) multiple levels of sampling with nested observations, (b) variables (i.e., antecedents, mediators, and outcomes) that occur at different levels, (c) constructs which represent shared unit characteristics which are measured through aggregation of individual responses, (d) randomization at the cluster level, and (e) both quantitative and qualitative analyses. Table 1 summarizes each characteristic and associated recommendations for implementation researchers; we envision it could be used as a simple planning tool or evaluative checklist. We also hope Table 1 encourages readers to use our eight characteristics as a whole, avoiding the problems associated with best practice misuse (e.g., cherry-picking specific sections to justify singular decisions while ignoring the others) [16].

**Table 1:** Summary of multilevel study characteristics and recommendations for implementation researchers

Characteristic	Recommendations	In-paper resources
**To conduct rigorous, high-quality multilevel implementation research...**		
*(1) Map and operationalize the specific multilevel context for defined populations and settings*	1a. Create and include a list or map of contextual levels most salient to the research question(s) and population(s) under study	▪ Table 2 ▪ Additional File 1 ▪ Glossary
*(2) Define and state the level of each construct under study*	2a. For each construct, define its substantive meaning and the level at which it resides/population unit with which it is associated 2b. For each construct, provide an explanation or “mini theory” that explains why the construct is assigned to its specific level/population unit	▪ Additional File 2 ▪ Glossary
*(3) Describe how constructs relate to each other within and across levels*	3a. Include a figure or narrative that describes the study’s theoretical model, including the level of each construct and the hypothesized relationships between constructs 3b. When hypothesized relationships cross levels, researchers should describe the processes through which higher-level antecedents influence lower-level consequents (i.e., top-down processes) or how lower-level antecedents shape higher-level consequents (i.e., bottom-up processes) 3c. Clarify each construct’s location in the study theoretical model relative to other constructs (e.g., is it an antecedent, mediator, consequent, primary or secondary endpoint, etc.)	▪ Fig. 1 ▪ Additional File 3 ▪ Glossary
*(4) Specify the temporal scope of each phenomenon at each relevant level*	4a. Provide a detailed explanation of the expected temporal dynamics within the study at each level, using visual aids as needed, to include the following: i. When investigators expect to observe change in each relevant outcome at each relevant level (e.g., of system- or organization-level implementation strategies) ii. How frequently and when constructs will be measured to capture these changes iii. How changes in outcomes at different levels align with each other in the research design iv. The theoretical rationale for these choices	▪ Additional File 4
*(5) Align measurement choices and construction of analytic variables with the levels of theories selected (and hypotheses generated, if applicable)*	5a. Align the levels of theory and measurement; for unit-level constructs, determine whether the construct is a global, shared, or configural property of the unit and use this to align measures with theory 5b. For shared constructs, address the following: i. Include a specific referent that indicates who and/or what is being rated ii. Effectively communicate these referents to participants in measurement instruments iii. Ensure respondents who are asked to report on shared constructs can report on them, and that they are the appropriate persons to ask iv. Provide evidence that individuals within a unit reflect (and can report on) a shared phenomenon or experience v. When shared constructs are measured quantitatively using individual responses, aggregate the individual responses into unit-level scores of shared constructs	▪ Additional File 5 ▪ Glossary
*(6) Use a sampling strategy consistent with the selected theories or research objectives and sufficiently large and variable to examine relationships at requisite levels*	6a. Design and justify a multilevel sampling plan, ensuring there is the following: i. A large enough sample at each level to rigorously test hypotheses or make theory-based inferences ii. Adequate *variability* within the sample *at each level* to rigorously test hypotheses or make theory-based inferences iii. Adequate *representativeness* of the achieved sample *at each level* (for quantitative) 6b. When reporting study findings for quantitative studies, include the following: i. The distribution and range of within-unit sample sizes ii. The distribution and range of within-unit response rates iii. A comparison of the characteristics of unit members who responded versus those who did not respond iv. The theoretical or empirical rationale for exclusion of units (as applicable)	▪ Additional File 6 ▪ Glossary
*(7) Align analytic approaches with the chosen theories (and hypotheses, if applicable), ensuring that they account for measurement dependencies and nested data structures*	7a. Directly acknowledge dependencies (i.e., correlated observations/nesting) within the proposed study design, articulate what analytic method has been selected to account for those dependencies, and provide a rationale for the choice of analytic method with reference to specific characteristics of the data and strengths of the selected method/model 7b. For quantitative, ensure that variables enter statistical models at the level warranted and scrutinize choices related to centering, standardization, and calculation of effect sizes to confirm they reflect the study’s multilevel design; for randomized studies, the variable representing randomization to condition (i.e., exposure) should enter the statistical model at the level at which randomization occurs 7c. Be transparent and thorough in reporting details of the analytic approach 7d. Consider developing and sharing crosswalks that specify research questions and justify the use of data collection tools and their accompanying analytic techniques, defining their multilevel purpose and (anticipated) contributions, including “explicit connections” or “intentional redundancies” among quantitative and qualitative approaches 7e. Consider making final analytic tools accessible to end users of multilevel research reports (e.g., qualitative interview guides, statistical code)	▪ Additional File 2 ▪ Additional File 7 ▪ Glossary
*(8) Ensure inferences are made at the appropriate level*	8a. Carefully craft and check language within research reports and presentations to ensure atomistic and ecological fallacies are not present	▪ Additional File 8 ▪ Glossary

## 1. Map and operationalize the specific multilevel context for defined populations and settings

### Rationale

Implementation﻿ studies are designed to make inferences about specific populations, which may consist of individuals, groups, organizations, or other systems that occur at specific levels in implementation contexts. Researchers should directly acknowledge these levels and their potential influence(s) on focal populations. Not doing so can lead to blind spots when conducting analyses and interpreting findings, and limit the generalizability of results. For example, a trial of an implementation strategy that identifies and equips clinical champions while focusing exclusively on clinic-level variables may ignore critical intra-clinic factors that may explain strategy effectiveness, such as variation in team-level leadership and characteristics of client/patient populations served [17].

### Our recommendation for implementation researchers

Create and include a list or map of contextual levels most salient to the research question(s) and population(s) under study. This map should justify the inclusion and exclusion of specific levels within the research design based on the research question and theory about how focal antecedents, processes, or outcomes relate to each other. Table 2 presents an example of levels that may (or may not) be included in an implementation study depending on the context and aligned with the Consolidated Framework for Implementation Research (CFIR) and the Exploration, Preparation, Implementation, Sustainment (EPIS) framework [18–21]﻿. Depending on the research questions, specific implementation studies may use only one or a few levels from this table (or some modification and expansion thereof). For more information on how to map the contextual levels within an implementation study, see Additional File 1.

**Table 2:** Conceptual model of levels in implementation research illustrated in CFIR and EPIS frameworks

CFIR / EPIS	Level name	Definition	Examples
Outer setting / Outer Context	Country^a^	A nation with its own government, occupying a specific territory (*Oxford languages dictionary*)	Denmark, USA, Honduras
	Health system^b^	An organization that includes at least one hospital and at least one group of physicians that provide comprehensive care (including primary and specialty care) who are connected with each other and with the hospital through common ownership or joint management (*U.S. Agency for Healthcare Research and Quality’s Compendium of US Health Systems, 2016*)	National Health System (UK), Kaiser Permanente (USA)
	Region^c^	An area, administrative district, or division of a country with definable characteristics or boundaries	District, county, parish, province, state
	Locality	An area or neighborhood that constitutes a subdivision of a larger social or political entity	Neighborhood, city, town, census tract
Inner setting / Inner context	Organization/agency	A group of people arranged within a formal legal structure (e.g., for-profit or not-for-profit) for the specific purpose of delivering healthcare. Exercises authority over departments/wards, clinics/practice sites, program/units, clinical teams, and/or providers	Hospital network; multistate organization delivering mental health services
	Department/ward	A subdivision of an organization with a specialized purpose, capacity, or workgroup(s)	Oncology unit within a hospital
	Clinic/practice/site	A single, specific, geographically distinct location in which, or from which, providers deliver health-related or behavior change interventions to a target population or populations. Serves as the operational center of programs/units, clinical teams, and/or providers	Primary care practice, outpatient mental health clinic, school
	Program/unit	A group of providers or clinical teams that deliver a specific, clearly defined health-related or behavior change intervention to a target patient population; programs are sub-units of higher-level entities such as clinics or organizations	Pediatric oncology ward in a children’s hospital
Individuals involved / Individual characteristics	Clinical team	One or more providers (including dyads) who directly co-deliver, or coordinate or share responsibility for delivery of, a health-related or behavior change intervention to a patient or patient population	Assertive Community Treatment team, Multisystemic Therapy team
	Provider	A single individual who delivers a health-related or behavior change intervention to the target patient	Clinician, prescriber, home visitor, peer-to-peer specialist, volunteer
	Patient^d^	The individual, or smallest group of individuals, targeted by the clinical intervention or policy to be implemented	Child with depression, adult experiencing HIV, family group
Process / timing within EPIS Phases	Observation/time	A single, specific point in time at which an implementation-relevant measurement is taken for either research or clinical purposes	Baseline status, posttreatment status, 3-month follow-up status

*CFIR* Consolidated Framework for Implementation Research [20]; *EPIS*  Exploration, Preparation, Implementation, Sustainment Framework [21]

^a^From a geopolitical and social standpoint, we acknowledge this list is not exhaustive; investigators should refine this framework for their unique implementation context. For example, there are levels that could be specified beyond “country.” We terminated our framework here because most implementation research questions limit their unit of analysis to nations or lower-level geopolitical units due to the significant influence of national governments on health and health policy; however, we acknowledge these categories are not exhaustive, and we encourage researchers to modify this framework as needed

^b^We note that health systems may span a single or multiple localities, regions, or nations

^c^Depending on the nature of the study, the level of region may need to be subdivided into smaller units, such as census tracts nested within counties, nested within states, and nested within subregions

^d^We note that the patient level may pertain to an individual, dyad, or family group, depending on the nature of the clinical intervention or policy under study. We also note that potential sublevels embedded within the patient level may need to be accounted for depending on the study design. For example, multiple children receiving care may be embedded within a single family

## 2. Define and state the level of each construct under study

### Rationale

After mapping the study’s multilevel context and associated populations, the next step is to define each construct and identify its level within the design. Clear construct definition is crucial because it provides the basis for the accurate construction of measures (Characteristic 5) and treatment of analytic variables (Characteristic 7) and supports appropriate interpretation of results (Characteristic 8) [7]. Constructs may include implementation determinants [22, 23], implementation strategies [10, 24], variables that are part of a mediation chain [25], variables that modify the effects of other antecedents (i.e., moderator, effect modifier), or implementation or clinical effectiveness outcomes [26].

### Our recommendation for implementation researchers

For each construct under study, define (1) its substantive meaning (i.e., what is it?) and (2) the level at which it resides and its associated population unit (e.g., does it occur at the level of patient, provider, team, clinic, organization?) [27]. For each variable, provide an explanation or “mini theory” that clarifies why the construct is assigned to its specific level and population unit [7]. For example, a study of hospitals might invoke the concept of organizational culture (defined following Schein as “a pattern of shared basic assumptions learned by a group as it solved its problems of external adaptation and internal integration, which has worked well enough to be considered valid and, therefore, to be taught to new members as the correct way to perceive, think, and feel in relation to those problems” ([28] p. 18]), assign it to the level of hospitals (i.e., culture is a characteristic of hospitals), and use organizational culture theory to explain how culture emerges at the hospital level. This definition and theory would guide measurement and analytic decisions. For more information on how to do this, see Additional File 2.

## 3. Describe how constructs relate to each other within and across levels

### Rationale

After defining each construct in terms of its meaning, level, and associated unit, investigators must clarify how study constructs relate to each other within and across levels. This step is essential to planning analyses. 

### Our recommendation for implementation researchers

**Fig. 1:**
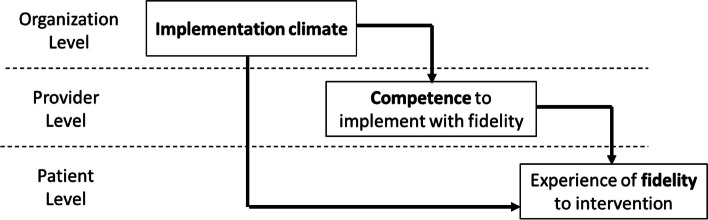
Example multilevel theoretical model. Note: In this example, the study tests the relationships between three constructs which occur at different levels of the implementation context. The researchers hypothesize that variation in implementation climate across organizations will explain variation in provider competence to implement a focal intervention with fidelity which in turn will explain variation in the extent to which patients experience fidelity to the focal intervention during the course of treatment

Research plans should include a figure or narrative that describes the study’s theoretical model, including the level of each construct and the hypothesized relationships between constructs. We also suggest that researchers specify each construct’s causal ordering in the study theoretical model (e.g., is it an antecedent, mediator, moderator, consequent, primary, or secondary endpoint) [27]. Figure 1 provides an example. Drawing on theory and prior research, researchers should provide a rationale for the proposed relationships within the model.

When hypothesized relationships cross levels, researchers should identify and describe the processes through which antecedents at higher levels influence consequents at lower levels (i.e., top-down processes) or how antecedents at lower levels shape consequents at higher levels (i.e., bottom-up processes). The description should include theoretical justification for each cross-level effect to be examined in the study. For example, if an investigator hypothesizes that increased clinic implementation climate, defined as employees’ shared perceptions of the extent to which their clinic expects, supports, and rewards the use of a specific intervention with high fidelity [29], will increase provider self-efficacy to deliver an intervention with high fidelity, this cross-level relationship implies an increase in the clinic means of provider self-efficacy, and the research plan should describe how and why that would occur. Alternatively, an investigator might hypothesize that high levels of implementation climate [29] will decrease the dispersion or variability of provider attitudes around their clinic means (i.e., the climate will increase the level of agreement among providers within a clinic). Since both of these variables (i.e., implementation climate and the magnitude of variability in attitudes) represent characteristics of the clinic, they occur at the same level, and the research plan should state how this same-level process would occur. For more information on how to do this, see Additional File 3.

## 4. Specify the temporal scope of each phenomenon at each relevant level

### Rationale

Rigorous multilevel implementation research requires thoughtful consideration of temporality (i.e., the sequence of events that unfold over calendar time) and pace of change (i.e., tempo or speed of change) as well as how these might differ across levels and align within a research design. People, organizations, and other systems change over time; however, the sequence or pace of change at one level may differ from that at another level [7]. For instance, an organization’s culture may be slow to change compared to specific aspects of policies or staffing. Additionally, organizations may change more quickly or slowly under different conditions. Externally imposed system reforms (e.g., funding shifts or policy changes) or crises (e.g., pandemics or natural disasters) may trigger more rapid change than internally planned changes. For instance, COVID-19 mitigation and other social distancing measures triggered a rapid shift from in-person service delivery to telehealth or other virtual platforms. Please see Additional File 4 for another example of this issue.

### Our recommendation for implementation researchers

We recommend that researchers provide a detailed explanation of the expected temporal dynamics within their study at each level, using visual aids as needed, which includes the following: (1) when investigators expect to observe change in each outcome at each relevant level (e.g., of system- or organization-level implementation strategies), (2) how frequently and when constructs will be measured to capture these changes, (3) how changes in outcomes at different levels align with each other in the research design, and (4) the theoretical rationale for these choices. Research plans should draw on relevant theory and report the expected direction, shape, frequency, and/or tempo of anticipated change in focal constructs, at each level and across levels, with decisions about measurement frequency and timing linked to these theoretical expectations. Measurement intervals and durations may differ at each level depending on the expected temporal dynamics and emergent issues. For more information on how to do this, see Additional File 4.

## 5. Align measurement choices and construction of analytic variables with the levels of theories selected (and hypotheses generated, if applicable)

### Rationale

The operationalization and measurement of variables must align with theory so that inferences about selected constructs accurately reflect target levels and populations. Put simply, measurement must align with the level of theory. By level of theory, we mean the level at which the construct has been defined (i.e., in Characteristic 2). An example of measurement-theory *mis*alignment is using individually varying scores to measure a theoretically shared organizational characteristic such as organizational climate. Ensuring measurement-theory alignment requires investigators to understand the theoretical assumptions embedded within each of the study’s constructs (e.g., organizational climate assumptions) and provide evidence that the measurements taken conform to those theoretical assumptions [7].

### Our recommendation for implementation researchers

Align the levels of theory and measurement. Such alignment is often most difficult for unit-level constructs; however, the organizational research literature offers a useful typology of categories of variables (global, shared, and configural) to aid investigators in this task [7]. G*lobal* constructs are those that originate at the unit level and represent objective, easily observable characteristics of the unit. Examples of global constructs include the type of hospital ward or unit (e.g., pediatric, intensive care) and the number of patients seen by the unit in a year. S*hared* constructs originate at the individual level but are shared across unit members [7]. An example of a shared construct is clinic implementation climate [29]. Note that even though clinic implementation climate originates at the provider level (i.e., in individual provider perceptions), it is conceptualized as a characteristic of the unit because it is a shared, contextual feature of the work environment. C*onfigural* constructs originate at the individual level and represent a pattern of individual characteristics within the unit [7]. Examples of configural constructs include variation in years of clinical experience on a team or diversity of professional roles within the team, or the optimal performance by a single member of the team. This typology directly informs the selection of appropriate measurement approaches and guides the type of validity evidence investigators should provide to demonstrate alignment between theories and measures. For example, investigators may need to provide evidence of within-clinic agreement on clinician perceptions of implementation climate in order to support the validity of the clinic implementation climate construct within their study [30]. For more information on how to align the levels of theory and measurement, see Additional File 5.

## 6. Use a sampling strategy consistent with the selected theories or research objectives and sufficiently large and variable to examine relationships at requisite levels

### Rationale

In multilevel studies, there are different sample sizes and sampling plans at each level of the design. For example, in a study of community health workers embedded within primary care clinics, the inferences drawn will be shaped by the samples of clinics and by the sample of workers within each clinic. As with all samples, investigators must attend to the number of participants necessary to generate appropriate statistical or theoretical inferences, the distribution of participants’ characteristics (to ensure adequate variability), and their representativeness of a target population. However, this logic applies separately to each level’s specific sample(s).

Special attention is often needed to ensure that the number and representativeness of participants within each higher-level unit are adequate to address the research questions and are aligned with the theoretical or conceptual model. For example, how representative of a clinic are participants’ responses if only two of ten workers complete study surveys? What is the minimum number of participants needed per clinic? What are the implications of variation across clinics in their within-clinic response rates?

### Our recommendation for implementation researchers

Given the considerations above, multilevel implementation studies should be designed to ensure there is (1) a large enough sample at each level to test hypotheses or make theoretical inferences rigorously, (2) adequate variability within the sample at each level to achieve these objectives, and (3) representativeness of the achieved sample at each level (for quantitative). To help readers of research reports assess these study characteristics, we recommend that quantitative multilevel implementation studies report the following: (1) the distribution and range of within-unit sample sizes, including a measure of central tendency (median/mean), dispersion (standard deviation), and minimum and maximum values (e.g., median, minimum, and maximum number of providers and/or patients per clinic); (2) the distribution and range of within-unit response rates (e.g., calculate the survey response rate within each clinic and report the mean, standard deviation, minimum, and maximum response rates); (3) a statistical comparison of the characteristics of unit members who responded versus nonresponders; and (4) the theoretical or empirical rationale for exclusion of units (e.g., on the basis of response rates or number of participants).

In qualitative studies, the goal is typically not to obtain a representative sample but to purposefully select cases or participants that meet preselected criteria that address the study’s research questions. Nonetheless, it is critical to ensure that investigators sample at all specified levels for analytic purposes, striving for sufficient sample sizes of the population units at each pertinent level and attending to consistencies, contradictions, and interconnections across levels. For example, in a study examining an organizational implementation strategy, investigators will likely be interested not just in what executives say about change processes related to an innovation’s uptake but also in triangulated data from first-level leaders (i.e., those who supervise providers) and direct service providers. Sampling at the different levels enables a more nuanced perspective on the interplay between levels and how they might influence each other. For more information on designing and justifying a quantitative or qualitative multilevel sampling plan, see Additional File 6.

## 7. Align analytic approaches with the chosen theories (and hypotheses, if applicable), ensuring that they account for measurement dependencies and nested data structures

### Rationale

Although there is no single best way to analyze data from multilevel implementation studies, investigators must ensure that analytic choices (1) account for the dependencies that arise in hierarchically sampled observations and (2) align with the study’s level(s) of theory and hypotheses or research aims [9]. This applies equally to quantitative, qualitative, and mixed-methods designs. Traditional quantitative data analytic approaches, such as ordinary least squares regression and *t*-tests, assume observations are independently sampled and thus uncorrelated. Statistical inferences are biased when this assumption is violated, as often occurs in multilevel designs where observations at a lower level (e.g., patient outcome scores) are nested within higher-level units (e.g., providers).

In qualitative studies, researchers can query the extent to which there is agreement or disagreement within levels (e.g., perceptions of leadership among a clinical team) and across levels (e.g., perceptions of leadership that vary between leader peer reports and subordinate reports of that leader) [31, 32]. Qualitative research can also help elucidate the kinds of complex nested relationships present within an implementation context [33] and can, therefore, provide valuable insight into what is most important to address related to levels of nesting. Qualitative research centered on process and the real-world interplay occurring across levels is especially useful for describing and contextualizing these dependencies while shedding light on how they likely operate to influence outcomes [34]. In ﻿addition, in the process of conducting qualitative research, we might identify new samples we may have not considered previously with participants who might have fresh insights into multilevel phenomena we are seeking to analyze.

### Our recommendation for implementation researchers

We recommend investigators directly acknowledge nesting and dependencies (i.e., correlated observations) within the proposed study design, articulate what analytic method has been selected to account for those features (or analytically demonstrate that the dependencies are not substantial enough to be a concern), and provide a rationale for the choice of analysis approach with reference to specific characteristics of the data and strengths of the selected model. For example, a quantitative study that measures fidelity to an intervention at the session level may need to account for the nesting of sessions within patients, nesting of patients within providers, and nesting of providers within clinics, depending on the specific sampling design and focus of the investigation. An analytic approach would be selected that addresses this nesting and a rationale provided for its use in this study.

For quantitative studies, we recommend that investigators ensure that variables enter statistical models at the level warranted and scrutinize choices related to centering, standardization, and calculation of effect sizes to confirm they reflect the study’s multilevel design [24, 35]. For randomized trials, the variable representing randomization to condition (i.e., exposure) should enter the statistical model at the level at which randomization occurs [36]; this often has significant implications for statistical power and sample size, particularly when the emphasis is on testing hypothesized mediators of implementation strategies’ effects [10].

The use of qualitative methods, such as participant observation, interviews, focus groups, and periodic reflections, is crucial to contextualizing and interpreting quantitative findings regarding dependencies and nesting while also offering in-depth insight into the range of anticipated and unanticipated factors emerging in real time that shape implementation processes and outcomes [37–40]. A variety of qualitative analytic techniques can be brought to bear in multilevel implementation research, including deductive techniques applying theoretical model constructs to support existing conceptualizations to test and validate theory and inductive techniques to generate new concepts, explanations, or theories from study data. Regardless of the approach taken, the key assessment criteria for analysis and interpretation of qualitative data center on ensuring a solid grasp of background issues and theory and a firm grounding in the data collected. Procedures that enhance the rigor and credibility of qualitative findings include investigating rival explanations pertinent to the phenomena of interest, accounting for disconfirming evidence and irregularities, and undertaking triangulation (within and across methods) [41–43]. The more data sources, the better. Triangulation practices typically entail summarizing analyses of all data sources and conducting side-by-side comparisons designed to corroborate and expand upon findings to create a complete or holistic picture of implementation processes and outcomes at the specified levels of interest [42].

Whatever analytic strategies are used to address multilevel designs in implementation research, we recommend investigators be transparent and thorough in reporting details of the analytic approach. We offer this general rule: as analytical complexity and decision points in an analysis increase, so should the level of description of the methods either in text or in a supplemental file. We also suggest investigators consider developing and sharing crosswalks that specify research questions and justify the use of data collection tools and their accompanying analytic techniques, defining their multilevel purpose and (anticipated) contributions, including “explicit connections” or “intentional redundancies” among quantitative and qualitative approaches [33]. Finally, we recommend that investigators make analytic tools (e.g., qualitative interview guides, statistical code) accessible to end users of multilevel research reports. For more information on how to create qualitative, quantitative, and mixed-methods multilevel analysis plans, see Additional File 7.

## 8. Ensure inferences are made at the appropriate level

### Rationale

When analyses are complete, investigators must ensure inferences are made at the appropriate level(s). Most researchers who discuss this issue [7, 44–46] focus on two primary fallacies regarding inferences from multilevel research: the atomistic fallacy and the ecological fallacy. The atomistic fallacy occurs when investigators analyze the association between variables at the individual level and then inappropriately make inferences about a higher level of analysis, such as groups or organizations [46]. Because the association between two variables at the individual level may differ from the association between the same or analogous variables at the group level, it is inappropriate to infer group-level relationships based on individual-level analyses [46]. For an implementation research example, see Additional File 8.

The ecological fallacy occurs when investigators conduct studies at a higher level of analysis (e.g., group, organization, or country) and inappropriately make inferences about lower-level units (e.g., individuals) [7]. Investigators should not use inferences based on data at the group level to substantiate relationships at lower levels of analysis for the same reason described for the atomistic fallacy. More specifically, the association between two variables at the group level may differ from the association between the same or analogous variables at the individual level. For an implementation research example, see Additional File 8. As Chan [45] highlighted, both fallacies are ultimately conceptual fallacies about interpreting results.

### Our recommendation for implementation researchers

Given these considerations, we recommend investigators carefully craft and check language within research reports and presentations to ensure atomistic and ecological fallacies are not present. Precise language is needed to describe the level of the constructs when discussing results. For instance, a conclusion like “higher readiness for change was associated higher fidelity” is vague about the level, as opposed to “higher unit-level readiness for change was associated with higher provider-level fidelity.” We suggest investigators increase their awareness of these fallacies and build in processes to check their assumptions when interpreting results from multilevel studies. We also recommend following Chan’s guidance to conduct multilevel analyses that appropriately account for variance within and between levels so that “analysis and interpretations can be aligned to avoid the conceptual problem of making inferences at the wrong level” ([45] p. 405).

## Conclusion

Implementation research is inherently multilevel. Building strong multilevel theories that explain the reality of implementation requires rigorous studies. Although the degree to which investigators account for this reality in their work may vary, as may the specific levels assessed in a particular study, we can meaningfully advance implementation science by articulating and enacting achievable standards of rigor for what constitutes high-quality multilevel research. We believe that shared standards of rigor can improve the quality, transparency, generalizability, and replicability of multilevel implementation research. In this paper, we took the first step in establishing and communicating such standards by distilling and translating key concepts from other fields (emphasizing the organizational sciences) for an implementation science audience.

Table 1 concisely summarizes our eight characteristics and associated recommendations for implementation researchers. Our eight characteristics are structured to guide the early conceptualization and grant-writing process. They are also intended to support investigators as they move through decision-making at each research phase — from research question formulation, variable selection and measurement, analysis, and the interpretation of findings. We hope these characteristics promote a common language and provide an initial template for planning for and evaluating the quality of multilevel implementation research. We also hope that acknowledging these characteristics will push the field forward in building testable multilevel theories that capture the complexities, and addresses the needs of implementation research.

These theories can be examined and tested using a range of designs and approaches. However, the complexity inherent in implementation research calls for other innovative approaches to understanding complex multilevel contexts. Systems science approaches (e.g., social network analysis, agent-based modeling, and systems dynamics) that account for nonlinearities, interdependencies, and cross-level phenomena have strong potential for expanding and testing multilevel theories [47]. However, even with these types of approaches, it is important to be clear about the ways in which within-level and across-level phenomena operate and interact. Our future work will delve into specific technical considerations and offer more detailed guidance for conducting multilevel research using traditional quantitative, qualitative, and mixed-methods approaches.

### Supplementary Information

**Additional file 1:** **Characteristic 1.** Map and operationalize the specific multilevel context for defined populations and settings.

**Additional file 2:** **Characteristic 2.** Define and state the level of each construct under study.

**Additional file 3:** **Characteristic 3.** Describe how constructs relate to each other within and across levels.

**Additional file 4:** **Characteristic 4.** Specify the temporal scope of each phenomenon at each relevant level.

**Additional file 5:** **Characteristic 5.** Align measurement choices and construction of analytic variables with the levels of theories selected (and hypotheses generated, if applicable).

**Additional file 6:** **Characteristic 6.** Use a sampling strategy consistent with the selected theories or research objectives and sufficiently large and variable to examine relationships at requisite levels.

**Additional file 7:** **Characteristic 7.** Align analytic approaches with the chosen theories (and hypotheses, if applicable), ensuring that they account for measurement dependencies and nested data structures.

**Additional file 8:** **Characteristic 8.** Ensure inferences are made at the appropriate level.

**Additional file 9:** **An Integrated Example.** The ASPIRE trial.

## Data Availability

Not applicable.

## Glossary

Characteristic #1: Map and operationalize the specific multilevel context for defined populations and settings.
Contextual levels: Context refers to the totality of space, time, and matter around a healthcare encounter. The combination of these terms indicates context has a hierarchical structure comprised of levels (using the definition of level provided below).
Level: A position within a hierarchical, nested structure. In implementation science, healthcare is delivered to patients, by providers, within organizations, and within larger systems. Each population in this chain (patients, providers, organizations, larger systems) is nested within another population. That is, multiple patients are cared for by a single provider, multiple providers work in a single organization, and multiple systems occur within a sociopolitical context such as a nation. Therefore, each population represents a level within the implementation context. By definition, levels are associated with specific populations.
Unit: A formal group of more than one person. Examples include teams, departments, divisions, wards, or clinics. A unit can also be conceptualized as an organization or system.
Characteristic #2: Define and state the level of each construct under study.
Analytic variable: The actual, observed value used to represent a construct within a quantitative or qualitative analysis.
Focal level: The level of the implementation context that is the center of attention for a particular research question. The focal level often refers to a location in the nested, contextual hierarchy at which a key variable of interest resides and/or is expected to affect.
Unit-level construct/property/characteristic: For the purposes of our paper, a unit-level construct/property/characteristic describes a feature, quality, or state of a unit. It may be observable or latent. A unit-level construct/property/characteristic can be further categorized as global, shared, or configural (see Characteristic 5 below). Examples for implementation research include clinic implementation climate, department safety climate, team demographic composition (e.g., in terms of workforce diversity), agency size, and site proximity to a university. See definitions for level and unit in Characteristic 1.
Characteristic #3: Describe how constructs relate to each other within and across levels.
Bottom-up processes: A sequence or series of events, or actions taken, in a specific order toward a specific outcome, which begin at a lower level and terminate at a higher level. An example is increased motivation among individual clinicians within a team to use a screening tool may lead to increased leader advocacy for funding for use of the tool (in response to the groundswell of support from clinicians), which may lead to increased funding available for the tool and greater reach of the tool to more patients within the organization.
Top-down processes: A sequence or series of events, or actions taken, in a specific order toward a specific outcome, which begin at a higher level and terminate at a lower level. An example is focused organizational implementation climate increasing individual clinicians’ self-efficacy to deliver an intervention with fidelity resulting in patients experiencing high fidelity to the intervention during service interactions.
Characteristic #5: Align measurement choices and construction of analytic variables with the levels of theories selected (and hypotheses generated, if applicable).
Aggregated: The process of combining responses from individuals to the unit level through some operation (often by taking the mean). For example, clinicians’ individual perceptions of their clinic’s climate could be aggregated by taking the mean of their responses to represent their shared experience (i.e., the unit climate).
Compilation variable: An operationalization or measurement of a unit-level construct that is derived from observations obtained from individual within a unit and represents the configuration or pattern of the individual responses or observations.
Compositional variable: An operationalization or measurement of a unit-level construct that is derived from observations obtained from multiple individuals within the unit who are believed to be affected by the construct and whose perceptions or experiences are believed to converge and coalesce around a shared experience. For example, organizational implementation climate is often measured by collecting individual perception ratings from providers within a unit, and the average of these scores is taken to represent the shared perception, i.e., focused climate, of the unit. Implementation researchers should provide evidence to support the validity of compositional variables when they are used. For example, an assessment of inter-rater agreement should be employed to show that providers within each unit agreed with each other on their perceptions of focused climate. This confirms that the climate perceptions were shared, and that the compositional variable of climate, which enters models at the unit-level, is indeed a shared characteristic of the unit.
Configural construct/configural unit property: A characteristic of a unit that represents the pattern, variability, or configuration of individuals’ characteristics or contributions within the unit. Examples include the level of diversity in a team’s years of experience or the network density of relationships among organization members. These properties emerge at the individual level but are not assumed to coalesce or converge among unit members; instead, individuals make distinct contributions to the pattern or configuration in the unit which combine in complex, nonlinear processes to generate the unit-level property. In defining configural properties, investigators should explain the processes by which unique individual contributions combine to form the unit-level characteristic. Operationalized measures of configural constructs are sometimes called compilation variables (see more above).
Global construct/global unit property: A characteristic of a unit that is material, descriptive, typically easily observable, and originates at the level of the unit. Group size or unit function is examples. Global characteristics do not have their basis in individuals’ (or lower-level units’) characteristics (or interactions), and thus, there is no possibility of within-unit variation.
Referent: The Oxford English dictionary defines referent as “the thing that a word or phrase denotes or stands for.” In the context of multilevel implementation research, we use this term to refer to the person or unit to which a measure applies. For example, if someone is being asked to rate “leadership” within their unit, the items should have a referent which indicates which leader they are being asked to rate. If someone is asked to rate climate within their work environment, the item should have a referent of which unit’s climate they are rating. For example, are they rating their immediate team, their whole clinic, or their whole organization?
Shared construct/shared unit property: A characteristic of a unit that is common to unit members based on the convergence or coalescence of individuals’ experiences, perceptions, attitudes, values, cognitions, affect, or behavior. Shared unit characteristics originate at the individual level and emerge as a unit characteristic as a function of attraction, selection, attrition, socialization, social interaction, shared sense-making, group adaption, leadership, and other psychological processes. Organizational culture and EBP implementation climate are examples. When implementation researchers incorporate shared unit characteristics into their studies, it is especially important that they specify the processes believed to generate high levels of within-group agreement and consistency across individuals as well as provide evidence that the characteristic in question is truly shared across individuals; demonstration of within-group agreement or convergence helps support the construct validity of shared unit-level constructs. Operationalized measures of shared constructs are sometimes called compositional variables (see more above).
Unit-level construct/property/characteristic: See definition in Characteristic 2 above.
Validity evidence: Very simply, “validity evidence” represents data and analyses brought to bear to show that a variable represents what it is supposed to represent. Messick (1989) defined validity as “an integrated evaluative judgment of the degree to which empirical evidence and theoretical rationales support the adequacy and appropriateness of inferences and actions based on test scores and other modes of assessment” (p. 13). The *Standards for Educational and Psychological Testing* of the American Educational Research Association, American Psychological Association, and National Council on Measurement in Education (1999) calls for investigators to generate evidence of the validity of inferences generated from measures. For a given set of items which comprise a measure, traditional types of validity evidence relate to content coverage, response processes, internal structure, and relations to other variables. In multilevel studies, it is important to provide validity evidence for compositional variables such as implementation climate, to show that they represent a shared characteristic of a unit.
Characteristic #6: Use a sampling strategy consistent with the selected theories or research objectives and sufficiently large and variable to examine relationships at requisite levels. Sampling plan — We use the definition of sampling plan offered by the US National Institute of Standards and Technology in its Engineering and Statistics Handbook: “a sampling plan is a detailed outline of which measurements will be taken at what times, on which material, in what manner, and by whom…Sampling plans should be designed in such a way that the resulting data will contain a representative sample of the parameters of interest and allow for all questions, as stated in the goals, to be answered.”
Characteristic #8: Ensure inferences are made at the appropriate level.
Atomistic fallacy: When investigators analyze the association between variables at the individual level and then inappropriately make inferences about a higher level of analysis, such as groups or organizations.
Ecological fallacy: When investigators conduct studies at a higher level of analysis (e.g., group, organization, or country) and inappropriately make inferences about lower levels units (e.g., individuals).
Level of analysis: The level within the implementation context of which research data are representative. For example, measures of team size represent the team level of analysis, and measures of individual provider self-efficacy represent the individual provider level of analysis.
