# Supplementary material for: Eight characteristics of rigorous multilevel implementation research: a step-by-step guide
Source: Implement Sci. 2023 Oct 23;18:52. doi: 10.1186/s13012-023-01302-2 (PMC10594828; doi:10.1186/s13012-023-01302-2)
Supplement: Supplementary file 1 — Additional file 1: Characteristic 1. Map and operationalize the specific multilevel context for defined populations and settings. [file 13012_2023_1302_MOESM1_ESM.docx]

**Additional File 1.**

***Characteristic 1:*** Map and operationalize the specific multilevel context for defined populations and settings.

***Guidance for creating a map of contextual levels and associated populations:***

Use Table 2 as a starting point. Table 2 (referenced in text, see separate document labeled Table 2) presents one way to map relevant levels and associated population units within multilevel implementation studies using CFIR as a guiding framework. For a similar but alternative approach to conceptualizing hierarchical levels within implementation contexts, see the framework by Harrison and Shortell [1]. Table 2 acknowledges the multilevel environment *outside* the organization and the multilevel context *within* the organization. **Importantly,** Table 2 is only an initial template that should be modified and expanded for specific studies. It intentionally does not include an exhaustive list of all potentially relevant levels. An example of how Table 2 may need to be modified is for implementation studies within school settings in the USA. Relevant levels in the school implementation context may include, for example: State, school district, school, and classroom, among others [2]. Other studies may need to consider and include levels such as industrial sector [3], client service area, formal and informal inter-organizational network [4–6], neighborhood, or legislative and judicial district.

General environment. Organizations are hierarchically nested within the ‘general environment’ of sociopolitical units. As depicted in Table 2, this could include levels such as nations, states, provinces, counties, and cities. The general environment might also be shaped by influences such as educational or professional bodies. Mapping the general environment (often described as the outer setting in implementation frameworks [4–6]) is important because understanding how an organization is situated within a particular policy and resource environment can uncover external forces that affect an organization’s opportunities, limitations, and priorities during the potentially long process of implementation and sustainment [7].

Intra-organizational units. Within an organization, work is often organized into hierarchically nested units as might be depicted in organizational charts [4–6]. These charts may depict departments, teams, and sub-units, typically organized by function or role and comprised of individual staff members. Mapping the internal organizational environment is critical for understanding whether and how resources, expectations, and work tasks are distributed and aligned. Examples of intra-organizational units that are relevant to implementation research include a unit organized by: the provision of an evidence-based practice (e.g., a team of clinicians providing Assertive Community Treatment), client population served (e.g., department serving children ages 0-5), disciplinary specialty (e.g., ob/gyn, surgery, psychiatry, or other department), how services are delivered (e.g., units delineated by service delivery location such as ambulatory care, schools, homes, and offices), employee type (e.g., physician, nurse, team of peer advocates), leadership level (e.g., upper management team), or funding source (e.g., county funded employee vs. non-county funded employee groups).

***Practical considerations:***

Oftentimes it is crucial to obtain input from implementation partners (e.g., system or organizational leaders, providers, participants in care) who can help map the implementation context and inform which levels should be addressed in the study design and analyses [3]. For guidance on how to conceptualize levels and multilevel influences on individuals within organizations, see work by Ferlie and Shortell [8] and Harrison and Shortell [1], which specify nested levels of healthcare systems. Applying a multilevel framework in research can generate unique insights about factors influencing change and implementation. For instance, Harrison & Shortell [1] illustrated how mapping pressures at each level of the system helped generate new insights about misalignment in external pressures from government regulations and internal priorities and resources, explaining why a change initiative did not achieve the expected impacts [9].

***Prompts to consider when creating your own map of contextual levels:***

When identifying and justifying which levels and units (external to the organization) could be relevant to the study:
□ Will environmental influences affect lower-level units (e.g., within the organization)? How will we account for these influences in our analysis and inferences?
□ What sources of theory and evidence support our prioritization of outer context levels and units? How will we report this information?

When identifying and justifying which hierarchically nested intra-organizational levels and units to include in the study:
□ What theoretical or practical basis is there for specifying the intra-organizational levels and units in our study?
□ Who in the organization should we consult to identify the most appropriate and relevant levels and units?
□ Are there any organizational records (e.g., organizational charts) we could draw upon?
□ Do we need to address the presence of informal structures (e.g., informal social groups) in the organization that may affect our implementation research questions? If so, how will we identify those structures and address their influence?

***Glossary terms for Characteristic 1:*** Level, Contextual levels, Unit

**References:**

1. Harrison MI, Shortell SM. Multi‐level analysis of the learning health system: Integrating contributions from research on organizations and implementation. Learn Health Syst. 2021;5.

2. Brookman-Frazee L, Chlebowski C, Suhrheinrich J, Finn N, Dickson KS, Aarons GA, et al. Characterizing shared and unique implementation influences in two community services systems for autism: applying the EPIS Framework to two large-scale autism intervention community effectiveness trials. Adm Policy Ment Health. 2020;47:176-187.

3. Kozlowski SWJ, Klein KJ. A multilevel approach to theory and research in organizations: Contextual, temporal, and emergent properties. In: Kozlowski SWJ, Klein KJ, editors. Multilevel theory, research, and methods in organizations: Foundations, extensions, and new directions. San Francisco, CA: Jossey-Bass; 2000. p. 3–90.

4. Aarons GA, Hurlburt M, Horwitz SM. Advancing a conceptual model of evidence-based practice implementation in public Service Sectors. Adm Policy Ment Health. 2011;38:4-23.

5. Damschroder LJ, Aron DC, Keith RE, Kirsh SR, Alexander JA, Lowery JC. Fostering implementation of health services research findings into practice: a consolidated framework for advancing implementation science. Implement Sci. 2009;4:50.

6. Moullin JC, Dickson KS, Stadnick NA, Rabin B, Aarons GA. Systematic review of the Exploration, Preparation, Implementation, Sustainment (EPIS) framework. Implement Sci. 2019;14:1.

7. Snell-Rood C, Trott Jaramillo E, Gunderson L, Hagadone S, Fettes D, A. Aarons G, et al. Enacting competition, capacity, and collaboration: performing neoliberalism in the U.S. in the era of evidence-based interventions. Crit Public Health. 2022;32:283–94.

8. Ferlie EB, Shortell SM. Improving the quality of health care in the United Kingdom and the United States: a framework for change. Milbank Q. 2001;79:281–315.

9. Leggat SG, Balding C. A qualitative study on the implementation of quality systems in Australian hospitals. Health Serv Manage Res. 2017;30:179–86.

**Three additional references that we recommend for Characteristic 1:**

Mathieu JE., Chen G. The etiology of the multilevel paradigm in management research. J of Management. 2011;37:610-641.

Gully SM, Phillips JM. On finding your level. In Humphrey SE, LeBreton JM, editors. The handbook of multilevel theory, measurement, and analysis. Washington, DC: American Psychological Association; 2019. p. 11-38.

Ostroff C. Contextualizing context in organizational research. In Humphrey SE, LeBreton JM, editors. The handbook of multilevel theory, measurement, and analysis. Washington, DC: American Psychological Association; 2019. p. 39-65.
