# Supplementary material for: Eight characteristics of rigorous multilevel implementation research: a step-by-step guide
Source: Implement Sci. 2023 Oct 23;18:52. doi: 10.1186/s13012-023-01302-2 (PMC10594828; doi:10.1186/s13012-023-01302-2)
Supplement: Supplementary file 2 — Additional file 2: Characteristic 2. Define and state the level of each construct under study. [file 13012_2023_1302_MOESM2_ESM.docx]

**Additional File 2.**

***Characteristic 2:*** Define and state the level of each construct under study.

***Guidance for assigning constructs to levels:***

Constructs should be assigned to levels on the basis of theory as applied to the specific study. For example, theory indicates focused organizational climates, such as implementation climate, are a unit-level characteristic (i.e., not a characteristic of a person) that can emerge at different levels (e.g., organization, site, team) because of shared experiences and interactions among members within the unit. Variables assessing focused climates should therefore be defined at a unit level (the unit being based on the study’s specific area of focus) with a rationale for how the climate emerges within that unit.

In randomized controlled trials, the condition variable (i.e., assignment to treatment versus control) is defined by the level of randomization (e.g., individual-randomized vs. cluster-randomized at a specific level) [1] and a rationale for the level of randomization should be provided. For example, in a study of a clinic-wide implementation strategy, randomization should occur at the clinic level to align with the strategy’s intended target (i.e., change in clinic characteristics) and to avoid contamination of lower-level outcomes such as patient symptoms. See [2] for guidance on determining the level of constructs within a research study.

When engaged in qualitative work, teams should be prepared to ask questions and conduct observations of the specified units at their appropriate levels, while making sure that data collection instruments are attentive of theoretical constructs. In terms of the oftentimes post-positivist orientation of implementation science, qualitative data collection tends to be influenced by conceptual models and their constructs [3]. At the same time, investigators should be open to findings that may not keep with existing models and constructs.

To aid this endeavor, we propose teams create crosswalks depicting how their qualitative data collection methods and instruments reflect specified units, levels, and constructs. For example, if an intervention is geared toward outcomes at the organizational level, investigators should ensure that qualitative instruments investigate change at this level. At the same time, individual experience can be illustrative of changes happening at other levels. The key is to document the interplay between levels, while discerning patterns occurring among individuals (e.g., those participating in one-on-one interviews) that suggest a higher-level change is happening. The specific data collection method will depend on the populations, levels, and outcomes of interest.

For example, when we are using the Dynamic Adaptation Process to make change happen in a school or school-based health center, we will do focus groups rather than individual interviews to efficiently tap into the perspectives of key folks involved in implementation, allowing them to think about and discuss their own views and experiences of this process and perceptions of outcomes in relation to those of others [4]. It is also important to note that when we select this method, the group comprises the unit of analysis rather than the individuals who take part in the group (although the referent of a particular focus group question, however, could still be a higher level unit such as the school).

***Practical considerations:*** Several resources from the organizational sciences provide guidance for developing mini-theories that explain why a construct should (or should not) reside at a certain level. See Chen, Mathieu, et al.[5], Tay et al. [6], Chen, Bliese et al. [7 ]and Jebb et al. [8]. Qualitative approaches may be useful for clarifying or confirming the level at which a particular construct is most salient to participants or most relevant to improving an implementation outcome (e.g., is climate most saliently an organizational or team level construct within this setting?) [9].

***Prompts to consider when you assign constructs to levels:***

When providing a definition for each construct:

□ Have we defined all relevant implementation determinants, strategies, mechanisms, mediators, moderators, pre-conditions, and outcomes that we plan to investigate?

When specifying the level at which each construct resides:
□ What levels are most important for my practice setting? Ex: policy, system, inter-organizational, organizational, team.
□ What are our sources of theory and evidence for determining what is most important? How will we report this information?

When determining if each unit-level construct describes a global, shared, or configural property:

□ How do we know the type of unit-level construct (global, shared, or configural)?
□ What are our sources of theory and evidence for determining what type of unit-level construct it is? How will we report this information?

When specifying the unit associated with each construct:
□ What formal units are important in our practice setting? Ex: units designated in the organizational chart.

□ What informal units are important in our practice setting? How will we learn about these units? Ex: a subgroup of clinicians who are socially connected.

□ What are our sources of theory and evidence for determining formal and informal units? How will we report this information?

***Glossary terms for Characteristic 2:*** Focal level, Analytic variable, Unit-level construct/ property/ characteristic

**References:**

1. Campbell MK, Piaggio G, Elbourne DR, Altman DG. Consort 2010 statement: extension to cluster randomised trials. BMJ. 2012;345:e5661–e5661.

2. Harrison MI, Shortell SM. Multi‐level analysis of the learning health system: Integrating contributions from research on organizations and implementation. Learn Health Syst. 2021;5.

3. Hamilton AB, Finley EP. Qualitative methods in implementation research: An introduction. Psychiatry Res. 2019;280:112516.

4. Patton M. Qualitative research & evaluation methods. 4th ed. Thousand Oaks, CA: Sage Publications, Inc.; 2015.

5. Chen G, Mathieu JE, Bliese PD. A framework for conducting multilevel construct validation. In F. J. Yammarino & F. Dansereau, editors. Research in multilevel issues: Multilevel issues in organizational behavior and processes. Oxford, UK: Elsevier; 2004. p. 273-303.

6. Tay L, Woo SE, Vermunt JK. A conceptual and methodological framework for psychometric isomorphism. Organ Res Methods. 2014;17:77–106.

7. Chen G, Bliese PD, Mathieu JE. Conceptual framework and statistical procedures for delineating and testing multilevel theories of homology. Organ Res Methods. 2005;8:375–409.

8. Jebb AT, Tay L, Ng V, Woo S. Construct validation in multilevel studies. In Humphrey, S. & LeBreton, J., editors. The handbook of multilevel theory, measurement, and analysis. Washington D.C.: American Psychological Association; 2019. p. 253–78.

9. Bernard HR, Wutich A, Ryan GW. Research Design I. Analyzing qualitative data: systematic approaches. 2nd ed. Thousand Oaks, CA: Sage Publications, Inc.; 2017.

**Two additional references that we recommend for Characteristic 2:**

González-Romá V, Hernández A. Conducting and evaluating multilevel studies: recommendations, resources, and a checklist. Organ Res Methods. 2022; doi:10.1177/10944281211060712.

Mathieu JE, Luciano MM. Multilevel emergence in work collectives. In Humphrey SE, LeBreton JM, editors. The handbook of multilevel theory, measurement, and analysis. Washington, DC: American Psychological Association; 2019. p. 163–186.
