# Supplementary material for: Eight characteristics of rigorous multilevel implementation research: a step-by-step guide
Source: Implement Sci. 2023 Oct 23;18:52. doi: 10.1186/s13012-023-01302-2 (PMC10594828; doi:10.1186/s13012-023-01302-2)
Supplement: Supplementary file 3 — Additional file 3: Characteristic 3. Describe how constructs relate to each other within and across levels. [file 13012_2023_1302_MOESM3_ESM.docx]

**Additional File 3.**

***Characteristic 3:*** Describe how constructs relate to each other within and across levels.

***Guidance for specifying theoretical relationships across levels:***

Theoretical connections between variables may be based on prior empirical research or ‘big T’ theories, which are formally specified and often previously tested (e.g., the theory of planned behavior [1]). Connections may also be based on ‘little t’ theories, which represent newer ideas or more organically developed propositions which have often not yet been formally tested. The essential point for researchers is to articulate how and why variation in a construct that occurs at one level of the design is expected to relate to variation in a construct occurring at a different level of the design.

Describing these theoretical processes forms the basis for selecting an appropriate analytic approach [2]. For example, if a researcher believes that a focused organizational implementation climate for a specific intervention will improve providers’ competence to use the intervention, the researcher should draw on organizational climate theory and theory about skill-development to explain how variation in the organizational characteristic of implementation climate will lead to changes in the organizational means of provider competence. Figure 1 shows a theoretical model that reflects these hypotheses. Qualitative analyses can expand, illustrate, confirm, or challenge the cross-level relationships observed between quantitatively assessed antecedent and outcome variables at different levels of the design.

**Figure 1. Theoretical model of organizational implementation climate, provider competence, and patient-level fidelity.**


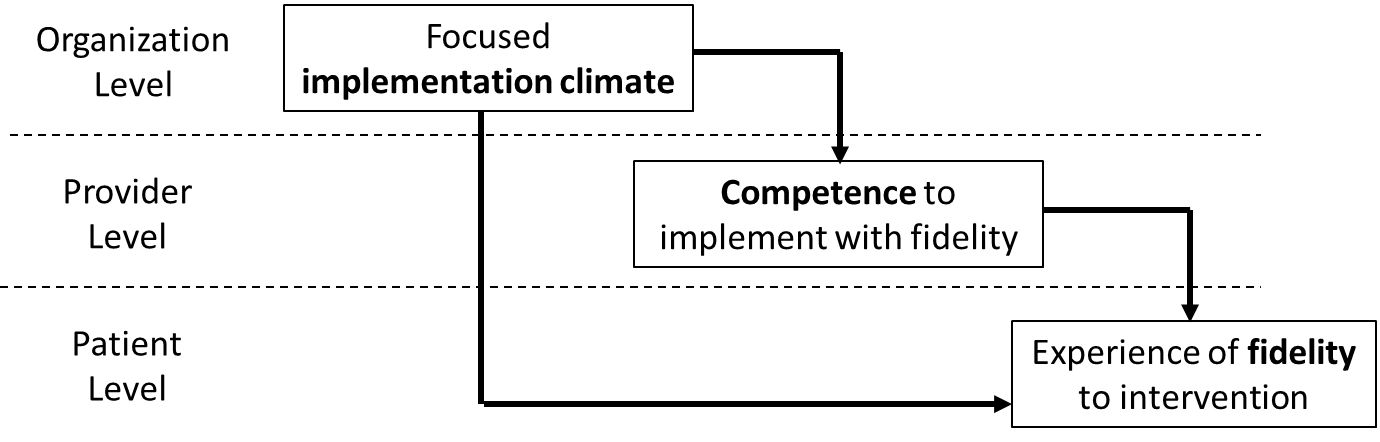

*Note:* In this example, the study tests the relationships between three constructs which occur at different levels of the implementation context. The researchers hypothesize that variation in implementation climate across organizations will explain variation in provider competence to implement a focal intervention with fidelity which in turn will explain variation in the extent to which patients experience fidelity to the intervention during the course of treatment.

***Practical considerations:*** Klein et. al.[3] and Rousseau [4] present typologies of relationships that may occur between variables at different levels within multilevel settings; these are useful for spurring investigators’ thinking in this area. Lewis et al. [5] offer guidelines for developing causal pathway diagrams which describe the relationships between constructs in implementation studies. It is important to note that when providing theoretical justification for each cross-level effect to be examined in the study, existing implementation process and determinant frameworks will likely not be sufficient as they typically do not specify causal and interactive relationships between levels. We suggest supplementing these types of implementation frameworks with ‘big T’ and ‘little t’ theorizing from your own field or discipline.

***Prompts to consider when specifying theoretical relationships across levels:***

When identifying relevant bottom-up (lower to higher-level) processes:
□ Are the lower and higher levels in each cross-level relationship specified?

□ What specific processes explain how thoughts, emotions, behaviors, or actions at a lower-level produce outcomes at a higher level?

□ What occurs to explain how a higher-level construct emerges from the interactions or combination of thoughts, emotions, or behaviors at a lower level?

When identifying relevant top-down (higher to lower-level) processes:
□ Are the lower and higher levels in each cross-level relationship specified?

□ How do constructs at higher levels influence the thoughts, perceptions, emotions, behaviors, experiences, or events at lower levels?

□ What is the process through which higher-level constructs influence the overall level or frequency of occurrence of thoughts, perceptions, emotions, behaviors, experiences, or events at the lower level?

When providing theoretical justification (can include more localized mini-theorizing) for each cross-level effect to be examined in the study:

□ What (big T or little t) theories and evidence explain the cross-level effects that interest us?

***Glossary terms for Characteristic 3:*** Top-down processes, Bottom-up processes

**References:**

1. Ajzen I. The theory of planned behavior. In: Lange PAM, Kruglanski AW, Higgins ET, editors. Handbook of theories of social psychology. London: Sage; 2012. p. 438–59.

2. Kozlowski SWJ, Klein KJ. A multilevel approach to theory and research in organizations: Contextual, temporal, and emergent properties. Multilevel theory, research, and methods in organizations: Foundations, extensions, and new directions . San Francisco, CA: Jossey-Bass; 2000. p. 3–90.

3. Klein KJ, Dansereau F, Hall RJ. Levels issues in theory development, data collection, and analysis. Academy of Management Review. 1994;19.

4. Rousseau DM. Issues of level in organizational research: Multi-level and cross-level perspectives. In: Cummings LL, Straw BM, editors. Research in organizational behavior . Greenwich, CT: JAI Press; 1985. p. 1–37.

5. Lewis CC, Klasnja P, Powell BJ, Lyon AR, Tuzzio L, Jones S, et al. From classification to causality: advancing understanding of mechanisms of change in implementation science. Front Public Health. 2018;6.

**Three additional references that we recommend for Characteristic 3:**

Bliese PD. Within-group agreement, non-independence, and reliability: Implications for data aggregation and analysis. In Klein KJ, Kozlowski SWJ, editors. Multilevel theory, research, and methods in organizations: Foundations, extensions, and new directions. New York: Jossey-Bass/Wiley; 2000. p. 349-381.

González-Romá V, Hernández A. Conducting and Evaluating Multilevel Studies: Recommendations, Resources, and a Checklist. Organ Res Methods. 2022; doi:10.1177/10944281211060712

Weiner BJ, Lewis MA, Clauser SB, Stitzenberg KB. In search of synergy: strategies for combining interventions at multiple levels. J. Natl. Cancer Inst., Monogr. 2012; 44:34-41.
