# Supplementary material for: Eight characteristics of rigorous multilevel implementation research: a step-by-step guide
Source: Implement Sci. 2023 Oct 23;18:52. doi: 10.1186/s13012-023-01302-2 (PMC10594828; doi:10.1186/s13012-023-01302-2)
Supplement: Supplementary file 4 — Additional file 4: Characteristic 4. Specify the temporal scope of each phenomenon at each relevant level. [file 13012_2023_1302_MOESM4_ESM.docx]

**Additional File 4.**

***Characteristic 4:*** Specify the temporal scope of each phenomenon at each relevant level.

***Guidance for*** ***deciding on, and explaining, the frequency and timing of measurements:***

Although we know that the external organizational environment tends to change more slowly than the internal organizational context, few studies have tracked changes over time in many constructs we include in multilevel implementation research. Therefore, there is limited theory or empirical evidence to guide decisions about when and how often implementation constructs at each level should be measured. For example, while implementation can lead to changes in clinicians’ communication patterns with their agency colleagues, there were minimal changes observed in a one-year study [1], raising questions about whether more time was needed to observe changes, or whether more substantial changes occurred earlier in the year, and clinicians returned to earlier patterns of communication afterwards. In these cases, partners who are directly involved in implementing the intervention or who have related experience might provide useful guidance. We recommend investigators rely on this localized tacit knowledge, when appropriate, through informal partner meetings. Formal qualitative study can also provide more systematic observations.

For example, qualitative research can help decipher the “noise,” or unanticipated factors emerging in real-time that shape implementation processes and outcomes but that might be missed by conventional quantitative measures, such as patient/client cultural perceptions of an intervention, provider non-adherence to research supported practices, or the complex social dynamics occurring at different levels of a system or organization [2,3]. Besides traditional interviews and focus groups, ethnographic methods might be especially well-suited for understanding the temporal scope of change at different levels of healthcare systems. Short-term ethnographic approaches might be practicable. For instance, Rapid Assessment Procedure Informed Clinical Ethnography (RAPICE), allows for observations by one or more trained ethnographers, leverages multidisciplinary team (including clinicians), allows for a fast-paced, yet iterative process of data collection, and reduces burden on participants [4]. Periodic reflections among implementation team members might also be useful [5]. Another option is to conduct pilot work with multiple measurements of key outcomes or endpoints across time to clarify the range of time it might take for a construct to change, and the point at which most units should have experienced change based on the exposure.

***Checklist of what to report in your research plan:***

□ When do you expect to observe change in each relevant outcome at each relevant level (e.g., of system- or organization-level implementation strategies)?
□ How frequently and when will you measure constructs to capture these changes?
□ How will changes in outcomes at different levels align with each other in the research design? For example, how long might it take for a leadership strategy to change supervisor’s behavior and what implications would that have for when we should see change in providers’ fidelity and ultimately in patient outcomes?
 *□* What are the theoretical rationales for these choices (formal ‘big T’ theories or informal ‘little t' theories)?

***Example of how the sequence or pace of change at one level may differ from that at another level:***

Consider a study in which a clinic-level implementation strategy is employed to improve clinic implementation leadership as a means of enhancing clinician fidelity to a targeted psychotherapy practice. The primary implementation outcome in this study is clinician fidelity and the primary clinical outcome is patient improvement in symptoms. Designing this study requires careful consideration of how the change process is expected to unfold across levels over time. It may take two to four months to change clinic leaders’ behaviors. Once clinic leadership is modified, it may take clinicians one to two months to change their behavior and increase fidelity in response to leadership changes. For individual patients receiving therapy, it may take three to six months of high-fidelity treatment before they begin experiencing symptom relief. Given this sequence, the implementation strategy might not be expected to improve fidelity until four months after completion of the leadership training and fidelity may not be expected to improve until 6-8 months after completion of the leadership training. All of these temporal dynamics must be taken into account in designing multilevel measurement and analysis plans.

***Practical considerations:*** Alexander and colleagues [6] provide guidance for addressing temporality in trials of multilevel cancer interventions. Collins and Graham [7] offer suggestions for timing of observations in longitudinal studies of drug and alcohol use. Glisson et al. [8] describe a trial that hypothesized when and how temporal dynamics were expected to shape the impact of an organization-level strategy on patient-level clinical outcomes. Williams et al. [9] describe a sequence of change in organizational and clinician constructs influencing clinician behavior.

***Prompts to consider when deciding and explaining the frequency and timing of measurements:***

When reporting the temporal assumptions for each cross-level effect in Characteristic 3:
□ Are there organizational characteristics (e.g., previous experience with EBP implementation or turnover rates) that could affect timing and/or rate of change?
□ Are there extra-organizational or extra-unit characteristics or phenomena that could affect timing or rate of change (e.g., seasonal patterns or a pandemic)?
□ How will timing issues affect our proposed study timeline and participant burden (e.g., duration of data collection activities)?

When deciding if time-scale differences across levels need to be acknowledged and/or reported:

□ If relevant, are bottom-up processes slower than top-down processes? Why or why not? How might this be important (practically and scientifically)?
□ Are other time scale differences between the levels important for understanding our implementation question(s)?
□ Who should we consult to identify and understand these differences (within and outside the organization)?

***Glossary terms for Characteristic 4:*** N/A

**References:**

1. Bunger AC, Lengnick-Hall R. Do learning collaboratives strengthen communication? A comparison of organizational team communication networks over time. Health Care Manage Rev. 2018;43:50–60.

2. Hohmann AA, Shear MK. Community-based intervention research: coping with the “noise” of real life in study design. Am. J. Psychiatry. 2002;159:201–7.

3. Getrich C, Heying S, Willging C, Waitzkin H. An ethnography of clinic “noise” in a community-based, promotora-centered mental health intervention. Soc Sci Med. 2007;65:319–30.

4. Palinkas LA, Zatzick D. Rapid Assessment Procedure Informed Clinical Ethnography (RAPICE) in pragmatic clinical trials of mental health services implementation: methods and applied case study. Adm Policy Ment Health. 2019;46:255–70.

5. Finley EP, Huynh AK, Farmer MM, Bean-Mayberry B, Moin T, Oishi SM, et al. Periodic reflections: a method of guided discussions for documenting implementation phenomena. BMC Med Res Methodol. 2018;18:153.

6. Alexander J, Prabhu Das I, Johnson TP. Time Issues in Multilevel Interventions for Cancer Treatment and Prevention. JNCI Monographs. 2012;44:42–8.

7. Collins LM, Graham JW. The effect of the timing and spacing of observations in longitudinal studies of tobacco and other drug use: temporal design considerations. Drug Alcohol Depend. 2002;68:85–96.

8. Glisson C, Williams NJ, Hemmelgarn A, Proctor E, Green P. Aligning organizational priorities with ARC to improve youth mental health service outcomes. J Consult Clin Psychol. 2016;84:713–25.

9. Williams NJ, Glisson C, Hemmelgarn A, Green P. Mechanisms of change in the ARC organizational strategy: increasing mental health clinicians’ EBP adoption through improved organizational culture and capacity. Adm Policy Ment Health. 2017;44:269–83.

**Three additional references that we recommend for Characteristic 4:**

Kozlowski SWJ, Klein KJ. A multilevel approach to theory and research in organizations: Contextual, temporal, and emergent properties. In: Kozlowski SWJ, Klein KJ, editors. Multilevel theory, research, and methods in organizations: Foundations, extensions, and new directions. San Francisco, CA: Jossey-Bass; 2000. p. 3–90.

Lang JW, Bliese PD, de Voogt A. Modeling consensus emergence in groups using longitudinal multilevel methods. Personnel Psychol. 2018;71:255-281.

Mathieu JE, Chen G. The etiology of the multilevel paradigm in management research. J of Management. 2011;37:610-641.
