# Supplementary material for: Eight characteristics of rigorous multilevel implementation research: a step-by-step guide
Source: Implement Sci. 2023 Oct 23;18:52. doi: 10.1186/s13012-023-01302-2 (PMC10594828; doi:10.1186/s13012-023-01302-2)
Supplement: Supplementary file 5 — Additional file 5: Characteristic 5. Align measurement choices and construction of analytic variables with the levels of theories selected (and hypotheses generated, if applicable). [file 13012_2023_1302_MOESM5_ESM.docx]

**Additional File 5.**

***Characteristic 5:*** Align measurement choices and construction of analytic variables with the levels of theories selected (and hypotheses generated, if applicable).

***Guidance for aligning measures with theory using the global, shared, configural typology:***

First, for measures of shared unit properties, such as unit leadership or unit climate, researchers must include a specific referent, which indicates who (e.g., which leader?) and/or what (e.g., expectations to use which clinical intervention?) is being rated. Moreover, these referents must be effectively communicated to participants in measurement instruments.

- *Implementation research example:* If clinicians are asked to rate ‘implementation leadership,’ the measure should clearly identify whose leadership they are rating (e.g., their immediate supervisor, the clinic’s medical director, or the organization’s executive?) and with regard to which clinical intervention or practice. Including the name or specific role of the leader to be rated is helpful. If clinicians are asked to rate ‘implementation climate,’ the measure should indicate what unit is being rated (e.g., their team, program, clinic, or organization) and what intervention is being rated. Implementation climate can vary depending on the level of the organization being assessed (e.g., team, clinic, full agency) and the clinical intervention being assessed. Referents for measures should be clearly articulated in the research plan and should align with the study theoretical model and hypotheses. For example, if an investigator wished to measure clinic climate for an intervention using an adapted version of a team climate measure, the investigator would need to articulate plans to modify the item referents for their study.

Second, researchers must ensure participants can report on shared unit constructs they are asked about and that the persons being asked are the ones whose (shared) perceptions matter.

- *Implementation research example:* Suppose providers interact minimally with a specific higher-level leader in an organization. In that case, it may be inappropriate to ask providers to assess that leader’s behavior [1]. Or consider a study in which the investigator is interested in understanding how team climate influences team members’ behavior. If team climate is measured solely on the basis of an outside observers’ perceptions, the focal construct (i.e., team members’ shared perceptions of their team’s climate) has not been assessed and the study cannot answer its focal question. Theory and input from partners and collaborators within the implementation context can guide the identification of which participants can accurately report on which constructs [2]. Empirical studies underscore the importance of these choices, given evidence of systematic differences between unit leaders and members regarding their perceptions of shared constructs such as culture and climate [3,4].

Third, when studying shared constructs such as culture or climate, researchers should provide evidence that reports from individuals within a unit reflect a shared phenomenon or experience [5].

- *Implementation research example:* Theory on organizational implementation climate indicates climate is a unit characteristic consisting of shared perceptions of providers who work together within a particular unit [6]. As such, researchers should conduct analyses that provide evidence to demonstrate that clinicians within each unit exhibited agreement on their climate ratings. The organizational science literature offers several accessible resources and tools for understanding and quantitatively calculating measures of interrater agreement and interrater reliability [7–9]. Examples include the rwg(j) and awg(j) indices and intraclass correlation coefficient (1) (ICC[1]). Implementation researchers should choose one or more of these approaches and use them to confirm that unit-level constructs represent shared experiences. Chan [5] provides a useful typology of composition models which describe how questions should be worded on measures depending on the nature of the shared unit property being assessed [9–11]. As an alternative to treating interrater agreement/reliability as ‘acceptable’ or ‘unacceptable,’ investigators can treat these outcomes as existing on a continuum. LeBreton et al. [7] provide suggestions for investigators interested in this approach.

Fourth [quantitative only], when variables representing shared constructs are measured using individual responses (e.g., individual clinician perceptions), researchers should aggregate the individual responses into unit-level scores and enter into statistical models at the unit level. This can be done multiple ways but should be explained and justified in the research plan.

- *Implementation research example:* Investigators may take the mean of clinicians’ individual climate ratings for each unit and enter the unit-level mean climate scores into analyses as observed variables (after demonstrating high inter-rater agreement within units). Alternatively, statistical procedures such as multilevel modeling could be used to combine individual climate ratings into latent unit means [12–14]. Lang et al. [15] describe procedures for modeling change in latent means of unit-level constructs over time. Their approach could be used, for example, to model change in the latent means of team psychological safety over time.

***Practical considerations:*** In our own work, we have found that qualitative research helpfully informs many of the key methodological decisions for measuring shared constructs like organizational climate. In school research, for example, qualitative research is useful for determining whether the school level or district level are the most salient referents for climate. This determination depends on how autonomous the school is and what the interconnections are like between school and district levels.

Qualitative approaches may be aimed at clarifying or confirming the level at which a particular construct is most salient to participants or relevant to improving an implementation outcome (e.g., is climate most saliently an organizational or team level construct within this setting?). Qualitative inquiry can also unearth new global, shared, or configural constructs that were not previously considered, especially if used during the exploratory phase of a study [16].

Investigators can also use qualitative data collection and analytic methods to shed light on why a selected theory may not fully explain the statistical data or to clarify unexpected results from a quantitative model. Qualitative analyses can elucidate how variables or processes may operate within or across organizational levels [17–19]. This can help to illuminate and subsequently address important gaps in quantitative measurement focused on a given level while contributing to the generation of new theoretical insights about phenomena occurring at that level (or across levels).

Regarding a practical consideration related to quantitative measures of leadership, an easy and oftentimes effective way to communicate the leader being rated (i.e., the referent) is to include the leader’s name in the item stems. For example, when rating implementation leadership, items might say: “*Dr. Williams* has developed a plan to facilitate implementation of EBP.” In this example, it is also important to specify the referent of the evidence-based practice being rated. Depending on the study’s focus, items may refer to general ‘evidence-based practice’ (in which case a definition should be provided) or they may refer to a specific clinical intervention (e.g., Parent-Child Interaction Therapy).

***Prompts to consider when aligning measures with theory:***

To help you clearly communicate to participants the referents for shared and configural constructs:
□ In all of our measurement materials (e.g., survey items, interview guides, instructions), is the referent for each item well-defined and consistent with the language and terms most familiar to the intended participants?
□ Are item referents aligned with the theoretical model and research questions?

When assessing and reporting on validity tests to support the construct validity of shared constructs:
□ If we plan to collect data from individuals about a shared construct, how will we know that we can meaningfully aggregate these individual-level reports to represent a shared higher-level phenomenon? What analytic approaches will generate validity evidence (e.g., rwg(j), awg(j), ICC(1), etc.)?
□ What theory can we use to justify our decision to aggregate individual-level reports to represent a shared higher-level phenomenon?
□ What practical insight and experience can we use to supplement formal theory (e.g., do these individuals meaningfully interact with each other)?

***Glossary terms for Characteristic 5:*** Unit-level construct/ property/ characteristic, Global construct/ global unit property, Shared construct/ shared unit property, Configural construct/ configural unit property, Compositional variable, Compilation variable, Referent, Aggregated, Validity evidence

**References:**

1. DeChurch LA, Hiller NJ, Murase T, Doty D, Salas E. Leadership across levels: levels of leaders and their levels of impact. Leadersh Q. 2010;21:1069–85.

2. Kozlowski SWJ, Klein KJ. A multilevel approach to theory and research in organizations: Contextual, temporal, and emergent properties. In: Kozlowski SWJ, Klein KJ, editors. Multilevel theory, research, and methods in organizations: Foundations, extensions, and new directions. San Francisco, CA: Jossey-Bass; 2000. p. 3–90.

3. Beidas RS, Williams NJ, Green PD, Aarons GA, Becker-Haimes EM, Evans AC, et al. Concordance between administrator and clinician ratings of organizational culture and climate. Adm Policy Ment Health. 2018;45:142-151.

4. Payne RL, Mansfield R. Relationships of Perceptions of Organizational Climate to Organizational Structure, Context, and Hierarchical Position. Adm Sci Q. 1973;18:515-526.

5. Chan D. Functional relations among constructs in the same content domain at different levels of analysis: a typology of composition models. J Appl Psychol. 1998;83:234-346.

6. Ehrhart MG, Schneider B, Macey WH. Organizational climate and culture. Routledge; 2013.

7. LeBreton JM, Moeller AN, Wittmer JLS. Data aggregation in multilevel research: best practice recommendations and tools for moving forward. J Bus Psychol. 2023;38:239–58.

8. Krasikova D V., LeBreton JM. Multilevel measurement: Agreement, reliability, and nonindependence. The handbook of multilevel theory, measurement, and analysis. Washington: American Psychological Association; 2019. p. 279–304.

9. LeBreton JM, Senter JL. Answers to 20 questions about interrater reliability and interrater agreement. Organ Res Methods. 2008;11.

10. Chan D. Multilevel research. In: Leong FTL, Austin JT, editors. The psychology research handbook. 2nd ed. Thousand Oaks, CA: Sage; 2006. p. 401–18.

11. Bliese PD. Within-group agreement, non-independence, and reliability: Implications for data aggregation and analyses. In: Klein KJ, Kozlowski SWJ, editors. Multilevel theory, research and methods in organizations: Foundations, extensions, and new directions. San Francisco, CA: Jossey-Bass; 2000. p. 349–81.

12. Rabe-Hesketh S, Skrondal A, Pickles A. Generalized multilevel structural equation modeling. Psychometrika. 2004;69:167–90.

13. Hox JJ. Multilevel regression and multilevel structural equation modeling. Oxford University Press; 2013.

14. Preacher KJ, Zyphur MJ, Zhang Z. A general multilevel SEM framework for assessing multilevel mediation. Psychol Methods. 2010;15.

15. Lang JWB, Bliese PD, Adler AB. Opening the black box: a multilevel framework for studying group processes. Adv Methods Pract Psychol Sci. 2019;2:271–87.

16. Bernard HR, Wutich A, Ryan GW. Research Design I. Analyzing Qualitative Data: Systematic Approaches . 2nd ed. Thousand Oaks, CA: Sage Publications, Inc.; 2017. p. 37–62.

17. Headley MG, Plano Clark VL. Multilevel mixed methods research designs: advancing a refined definition. J Mix Methods Res. 2020;14:145–63.

18. Bash KL, Howell Smith MC, Trantham PS. A systematic methodological review of hierarchical linear modeling in mixed methods research. J Mix Methods Res. 2021;15:190–211.

19. Aguinis H, Molina-Azorín JF. Using multilevel modeling and mixed methods to make theoretical progress in microfoundations for strategy research. Strateg Organ. 2015;13:353–64.

**Three additional references that we recommend for Characteristic 5:**

Jebb AT, Tay L, Ng V, Woo S. Construct validation in multilevel studies. In Humphrey, S. & LeBreton, J., editors. The handbook of multilevel theory, measurement, and analysis. Washington D.C.: American Psychological Association; 2019. p. 253–78.

Klein KJ, Conn AB, Smith DB, Sorra JS. Is everyone in agreement? An exploration of within-group agreement in employee perceptions of the work environment. J Applied Psychol. 2001; 86:3-16.

Wallace JC, Edwards BD, Paul J, Burke M, Christian M, Eissa G. Change the referent? A meta-analytic investigation of direct and referent-shift consensus models for organizational climate. J of Management. 2016; 42:838-861.
