# Supplementary material for: Eight characteristics of rigorous multilevel implementation research: a step-by-step guide
Source: Implement Sci. 2023 Oct 23;18:52. doi: 10.1186/s13012-023-01302-2 (PMC10594828; doi:10.1186/s13012-023-01302-2)
Supplement: Supplementary file 6 — Additional file 6: Characteristic 6. Use a sampling strategy consistent with the selected theories or research objectives and sufficiently large and variable to examine relationships at requisite levels. [file 13012_2023_1302_MOESM6_ESM.docx]

**Additional File 6.**

***Characteristic 6:*** Use a sampling strategy consistent with the selected theories or research objectives and sufficiently large and variable to examine relationships at requisite levels.

***Guidance for designing and justifying a multilevel sampling plan:***

Careful consideration of Characteristics 3-5 can help investigators determine the strengths and weaknesses of various sampling choices.

- Characteristic 3: Sample size and sufficient representation of the relevant is essential to studying the cross-level effects discussed in Characteristic 3. Without a sufficiently large and variable sample at each level, it is difficult to observe relationships among constructs.
- Characteristic 4: Issues of temporal scope affect sampling decisions in that many organizations involved in implementation studies experience turnover. Suppose investigators intend to detect changes in a configural unit construct such as the highest level of competence in delivering a clinical intervention within an Assertive Community Treatment team. In this case, they must focus on two aspects of the within-team sample collected at different time points.
  - First, are all (or almost all) members of the team surveyed at each time point so that there is reasonable confidence that the member with the highest competence level to deliver the intervention is represented in the sample? If not, this compromises inference.
  - Second, are the set of participants within a team the same across time points, or are there essentially two different teams assessed at Time 1 and Time 2 because of team member attrition? If so, theories and hypothesis tests may still be valid (indeed, some theories specifically address this contingency, see [1]. However, it remains incumbent upon investigators to describe how and why such tests remain appropriate [2].
- Characteristic 5: Will the targeted sample produce valid measures of the focal constructs, given those construct’s properties (i.e., shared, configural, and global)?
- Characteristic 5: Because shared and configural constructs originate at the individual level, investigators need to consider the representativeness of the samples within each unit. Research plans with a quantitative component should include procedures to track response rates within each unit as well as actions to ensure these are as high as possible. Units that lack sufficient sample size or sufficient responsiveness may compromise inferences.

In qualitative studies, sampling is guided closely by the investigator’s epistemological perspective underlying the design. Relevant for implementation studies is a traditional scientific approach grounded in deductive (e.g., theories, models, and frameworks) and inductive (e.g., grounded theory) approaches. Here, the goal of sampling is to develop theory-based generalizations through "rigorous and systematic comparisons of observed patterns with theoretical propositions" [3]. Qualitative research is thus used to generate, validate, or test theory.

If working from a realist standpoint, generalizations depend on purposive theoretical sampling that clarifies how the selected sample relates to a wider array of implementation settings or population units. If a social construction approach informs the work, the aim is to understand and compare the "reflective, experiential, and socially shared generalizations" within the population unit(s), showcasing the depth and detail derived from the data they provide[3]. A pragmatic perspective would involve developing modest, logical, problem-oriented speculations and extrapolations based on the likely applicability of findings to similar but not identical implementation contexts. The focus, from a pragmatic perspective, is on determining the practical value of what has been learned and how it can guide future actions, i.e., implementation processes. Drawing on information-rich cases is also useful when assessing applicability of findings to other related contexts[4,5].

Guided by the above, sample sizes for qualitative studies will often be determined based on saturation of content (i.e., findings repeat) as data are analyzed, ideally soon after they are collected. Relevant qualitative sampling approaches for implementation research include[3,6]:

1. **Criterion sampling** using preselected criteria, such as high provider adherence/fidelity to an intervention protocol or low patient satisfaction. Or, for example, perhaps we are interested in the specific experiences of all different types of leaders in an organization. Our goal then would be to define the types and levels of leadership we are most interested in understanding and then identify participants who represent each identified leadership type and level. Our theoretical and conceptual model will likely shape this approach and lend itself to triangulation and formal comparison to assess what is unique and different to leadership experiences across types and levels.
2. **Maximum variation sampling** to select cases (e.g., an implementation setting) representing variations on specified aspects of phenomena of interest. Here, we are interested in identifying central themes that cut across variation while preserving each case's unique details. An example might include sampling implementation sites across rural and urban geographies to analyze and compare how implementation barriers arise and are addressed across localities to produce generalizable findings.
3. **Extreme or deviant case sampling** (also known as "outlier sampling"), where we select cases that differ substantially from the majority, i.e., outstanding successes and notable failures, for example, in the achievement of an implementation outcome like penetration.
4. **Homogenous sampling**, where we select similar cases to explore an issue in-depth (this is the approach most commonly used in focus group sampling). An example might be sampling mental health providers who deliver the same intervention across multiple sites of a large federally qualified health center.
5. **Critical case sampling** where we select for "information rich" cases so that we can do a deep dive into learning about the phenomenon of interest. The case is selected because the evidence generated from it allows logical generalization and application to other very similar cases. The underlying idea is that if it’s true of this case, it’s likely to be true of all other cases in the same category; colloquially, ‘if it works here, it’ll work anywhere.’

***Practical considerations:*** Regardless of the qualitative sampling strategy and to support generalizability, we encourage implementation researchers: (a) to ensure that participants are thoughtfully sampled to get at characteristics of implementation settings, events, and processes relevant to our theoretical/conceptual model and research questions of interest, and (b) try not to rely on one method alone. For example, people might tell an interviewer what they do, but ethnographic observations might reveal something different. We also want to avoid sampling so narrowly that we miss out on collecting contrasting and comparative information to increase confidence, credibility, and the chances that findings may generalize to other implementation settings and populations.

General guidelines about sample size for multilevel research is reviewed by Hoyle and Gottfredson [7], González-Romá and Hernández [8], Hox and McNeish [9], and McNeish and Stapleton [10]. The PowerUpR tool (available at <https://powerupr.shinyapps.io/index/> ) by Bulus et al. ([https://CRAN.R-project.org/package=PowerUpR](https://cran.r-project.org/package=PowerUpR).) is extremely useful for determining sample size in multilevel trials; it addresses designs examining fixed effects, moderation, and mediation. Newman and Sin [11] and LeBreton et al.[12] offer guidance for dealing with situations in which units have missing responses or low levels of agreement on shared unit-level constructs such as climate.

To ensure high response rates within each participating unit, we have found it helpful in our work to systematically track response rates within each unit during active data collection. Monitoring not just the overall response but the response within each unit allows us to respond quickly and give extra attention and effort to units that are not achieving representative samples. For example, researchers could follow up with those units to better understand the barriers to participation. Additional recruitment visits to specific sites with low response rates may be helpful to provide additional reminders and opportunities for participation. Alternative times or formats for data collection can also be considered to increase representation and participation.

***Prompts to consider when designing and justifying your multilevel sampling plan:***

□ How many levels should we include in our analysis? Is each level worth the extra data collection burden?
□ Is the sample large enough at each level to assess statistical and/or theoretical inferences rigorously?
□ Will there be adequate variability in the sample (e.g., number of individuals, teams, or organizations) for each level to assess statistical and/or theoretical inferences rigorously?
□ Is there adequate representativeness of the target population at each level?
□ Are there any issues related to temporal scope (Characteristic 4) that we need to account for, such as how quickly change may occur at different levels?

***Checklist of what to report your quantitative sampling plan:***

□ The distribution and range of within-unit sample sizes, including a measure of central tendency (median/ mean), dispersion (standard deviation), and minimum and maximum values (e.g., median, minimum, and maximum number of providers and/or patients per clinic).

□ When applicable, the distribution and range of within-unit response rates (e.g., calculate the survey response rate within each clinic and report the mean, standard deviation, minimum, and maximum response rates).

□ When possible, a statistical comparison of the characteristics of unit members who responded versus those who did not respond.

□ If units are excluded on the basis of response rates or number of participants, the theoretical or empirical rationale for these choices.

***Glossary terms for Characteristic 6:*** Sampling plan

**References:**

1. Williams NJ, Ehrhart MG, Aarons GA, Marcus SC, Beidas RS. Linking molar organizational climate and strategic implementation climate to clinicians’ use of evidence-based psychotherapy techniques: cross-sectional and lagged analyses from a 2-year observational study. Implement Sci. 2018;13:85.

2. Cafri G, Hedeker D, Aarons GA. An introduction and integration of cross-classified, multiple membership, and dynamic group random-effects models. Psychol Methods. 2015;20:407–21.

3. Patton M. Qualitative research & evaluation methods. 4th ed. Thousand Oaks, CA: Sage Publications, Inc.; 2015. p. 719

4. Stake RE. Multiple case study analysis. The Guilford Press; 2006.

5. Kim B, Sullivan JL, Ritchie MJ, Connolly SL, Drummond KL, Miller CJ, et al. Comparing variations in implementation processes and influences across multiple sites: What works, for whom, and how? Psychiatry Res. 2020;283.

6. Bernard HR, Wutich A, Ryan GW. Research design I. Analyzing qualitative data: systematic approaches. 2nd ed. Thousand Oaks, CA: Sage Publications, Inc.; 2017. p. 37–62.

7. Hoyle RH, Gottfredson NC. Sample size considerations in prevention research applications of multilevel modeling and structural equation modeling. Prev Sci. 2015;16:987–96.

8. González-Romá V, Hernández A. Multilevel modeling: research-based lessons for substantive researchers. Annu. Rev. Organ. 2017;4:183–210.

9. Hox J, McNeish D. Small samples in multilevel modeling. Small sample size solutions. London: Routledge; 2020. p. 215–25.

10. McNeish DM, Stapleton LM. The effect of small sample size on two-level model estimates: a review and illustration. Educ Psychol Rev. 2016;28:295–314.

11. Newman DA, Sin H-P. How do missing data bias estimates of within-group agreement? Sensitivity of *SD _WG_ , CV _WG_ , r _WG(J)_ , r _WG(J)_* * , and ICC to systematic nonresponse. Organ Res Methods. 2009;12:113–47.

12. LeBreton JM, Moeller AN, Wittmer JLS. Data aggregation in multilevel research: best practice recommendations and tools for moving forward. J Bus Psychol. 2023;38:239–58.

**Three additional references that we recommend for Characteristic 6:**

Dong, N., & Maynard, R. PowerUp!: A tool for calculating minimum detectable effect sizes and minimum required sample sizes for experimental and quasi-experimental design studies. J Res Educ Eff. 2013;6:24-67.

Scherbaum, C. A., & Pesner, E. Power analysis for multilevel research. In Humphrey, S. & LeBreton, J., editors. The handbook of multilevel theory, measurement, and analysis. Washington D.C.: American Psychological Association; 2019. p. 329–352.

Williams NJ, Preacher KJ, Allison PD, Mandell DS, Marcus SC. Required sample size to detect mediation in 3-level implementation studies. Implement Sci. 2022;17:1.
