# Supplementary material for: Eight characteristics of rigorous multilevel implementation research: a step-by-step guide
Source: Implement Sci. 2023 Oct 23;18:52. doi: 10.1186/s13012-023-01302-2 (PMC10594828; doi:10.1186/s13012-023-01302-2)
Supplement: Supplementary file 7 — Additional file 7: Characteristic 7. Align analytic approaches with the chosen theories (and hypotheses, if applicable), ensuring that they account for measurement dependencies and nested data structures. [file 13012_2023_1302_MOESM7_ESM.docx]

**Additional File 7.**

***Characteristic 7:*** Align analytic approaches with the chosen theories (and hypotheses, if applicable), ensuring that they account for measurement dependencies and nested data structures.

***Guidance for planning multilevel analyses:*** Because of the breadth of this characteristic, we offer separate considerations for quantitative and qualitative/ mixed methods analysis plans.

Considerations for qualitative/ mixed methods multilevel analysis plans:

Mixed-method designs that begin with qualitative research (i.e., exploratory-sequential designs) are well suited for elucidating the elements and relationships in complex contexts that may not be fully known at the outset of the study, so that these are then appropriately represented in quantitative analyses [1,2].

- *Implementation research example:* The Dynamic Adaptation Process calls for multilevel assessment during the exploration phase of the Exploration, Preparation, Implementation, Sustainment (EPIS) framework to inform what levels of influence might be important in progressing through stages in the implementation process [3]. This type of approach can aid in identifying and elucidating important dependencies within the implementation context [4]. As a study progresses, qualitative research can help investigators test and contextualize the assumptions underlying their theory and hypotheses, and prompt the development of new hypotheses that were not previously considered [5,6].

Triangulation with qualitative data is also useful for further explaining multilevel quantitative results [1,2,6,7]. Qualitative studies have the potential to clarify key constructs, the levels of the system at which they operate, the direction of causality, and how we might want to treat them in the statistical analyses [4].

- *Implementation research example*: Social relationships among clinicians in their environment can shape their individual attitudes toward a new practice reflecting top-down causal mechanisms where social influence can shape attitudes [8]. Alternatively, clinicians often seek advice or discuss their work with peers who share similar attitudes reflecting a bottom-up selection mechanism where individuals’ attitudes shape relationships in the larger environment.

Designing a triangulation strategy for qualitative data generally entails summarizing analyses of all data sources (within or across methods) and conducting side-by-side comparisons to create a complete picture of the implementation processes and outcomes at the levels of interest. The theoretical/conceptual model is often useful for organizing triangulation (e.g., creating matrices to formally compare what happens at different levels or with varying population units across implementation phases). During triangulation, consideration should be given to each type of analysis on its own terms and how results differ or converge. Data can also be merged by (a) linking databases and (b) embedding one within the other so that each plays a supportive role. By using a process like Joint Display analysis [9,10], the results of each dataset are placed side-by-side to examine (1) convergence (do results provide the same answer to the same question); (2) expansion (are unanticipated findings of one dataset explained by another); and (3) complementarity (does embedding results of the qualitative analysis in the quantitative dataset help contextualize results [5,11]). Regardless of the specific qualitative analytic approach, investigators should adhere to standards for rigor, including credibility and authenticity, dependability and auditability, confirmability and objectivity, external validity, transferability, and fittingness, and utilization and application [12,13]

***Practical considerations:*** Qualitative researchers may wish to use procedural assessment tools, such as the Consolidated criteria for reporting qualitative research (COREQ) (<https://academic.oup.com/intqhc/article/19/6/349/1791966>). However, we note that use of such checklists is contested, e.g.,[14].

Considerations for quantitative multilevel analysis plans:

Traditional quantitative approaches to data analysis, such as ordinary least squares regression and t-tests, assume observations are sampled independently and thus are uncorrelated [15]. This assumption is necessary to generate accurate statistical inferences from the analyses; however, it is often violated when sampling occurs within multilevel contexts where observations at a lower level (e.g., patients) are nested within higher-level units (e.g., providers). Observations nested within the same unit are often correlated with one another and as such, investigators must use analytic techniques that account for this correlation.

- *Implementation research example*: Fidelity to an intervention experienced by patients of one provider are more likely to be similar to each other than they are to those of patients served by another provider.

Examples of analytic techniques that properly account for the correlation of nested observations include mixed effects models [16–18], generalized estimating equations [19,20], multilevel structural equation modeling [21,22], and fixed effects models for longitudinal designs [23], among others. Complex nesting scenarios in which a single individual is a member of multiple higher-level units (i.e., multiple membership) or moves from one higher-level unit to another during a study (i.e., cross-classification) also necessitate specialized analytic techniques [24]. We recommend investigators select an approach appropriate to their design and hypotheses.

We also recommend implementation researchers ensure that variables enter statistical models at the level warranted, and scrutinize choices related to centering, standardization, and calculation of effect sizes to confirm they reflect the study’s multilevel design [25,26]. For randomized studies, the variable representing randomization to condition (i.e., exposure) should enter the statistical model at the level at which randomization occurs [27]; this often has significant implications for statistical power and sample size, particularly when the emphasis is on testing hypothesized mediators of implementation strategies’ effects [28].

- *Implementation research example*: Stanhope et al. [29] provide an example of analyzing a cluster randomized controlled implementation trial where careful attention was paid to ensuring variables entered the model at the correct level. In their trial, 14 clinic sites were randomized to either (1) training in Person-Centered Care Planning (PCCP) using a train-the-trainer approach (N=7; experimental condition), or (2) service planning as usual (N=7; control condition). Data on PCCP fidelity was obtained via chart review of service plans. Fidelity scores were generated for a random sample of charts at three different time points. At each time point, chart fidelity scores were averaged across service plans within each time and within each team, making them time-varying, team-level fidelity scores. These were then modeled in a 3-level linear mixed model with time at level 1, team at level 2, and clinic at level 3. Randomization to condition entered the model at level 3 because clinics were randomized. Most control variables (covariates) pertained to the team level and thus entered the model at that level. Covariates were grandmean centered to address differences in case mix across teams and clinics.

***Practical considerations:*** González-Romá and Hernández [30] offer a useful checklist for investigators to address quantitative analytic issues in multilevel analysis. Hedeker and Gibbons[31], McNeish et al. [32], and Hubbard et al. [33] provide guidance for selecting between generalized estimating equations, mixed effects models, and other analytic approaches for clustered data. Allison [23] describes powerful fixed effects models for inference with longitudinal data.

***Prompts to consider when developing your multilevel analysis plan:***

When identifying potential dependencies in the data and selecting an analytic approach that accounts for them:
□ To what extent are observations in our study correlated with, or dependent on, each other because of how they are sampled (e.g., patients attending the same practice are served by the same physician and live in the same geographic area, so their health outcomes are likely to be correlated)?
□ If we have observations nested within higher-level units, how many higher-level units are there? What are the implications for addressing this situation in the analysis?
□ [Quantitative only] Should correlated observations be addressed using a statistical model or by aggregating them to a higher level (e.g., generate a single fidelity score for a patient based on multiple observations over the course of treatment)?
□ [Quantitative only] How do the theoretical assumptions of various statistical models (e.g., generalized estimating equations, mixed effects models) align or not align with our data?

When ensuring that analytic approaches properly translate and operationalize cross-level relationships into same-level relationships that offer appropriate inferences:
□ How do our analyses account for the within-unit and between-unit variance in independent and dependent variables?
□ How do these analytical decisions align with our research questions and hypotheses?

[Quantitative only] When ensuring that analytic choices regarding centering, standardization, and effect size account for the study hypotheses and design:
□ What centering and standardization choices have we made about our analytic variables?
□ What are their substantive implications for interpreting the analyses? Are these choices aligned with our selected theory?

***Checklist to use to evaluate the multilevel analysis plan you created:***

□ Did we directly acknowledge dependencies (i.e., correlated observations/ nesting) within the proposed study design?

□ Did we ensure that analytic choices account for the dependencies that arise in hierarchically sampled, nested observations (i.e., within-unit correlations)?

□ Did we ensure that analytic choices align with the study’s level(s) of theory and hypotheses?

□ [Quantitative only] Did we articulate what statistical method has been selected to account for dependencies among observations and did we provide a rationale for the choice of model with reference to specific characteristics of the data and strengths of the selected model?

□ [Quantitative only] Did we ensure that variables enter the statistical models at the level warranted and scrutinize our choices related to centering, standardization, and calculation of effect sizes to confirm they reflect the study’s multilevel design?

□ Are we transparent and thorough in reporting details of our selected multilevel analytic approach in project documents (e.g., study protocol, grant proposals, manuscripts)?

□ How will we make our analytic tools (e.g., qualitative interview guides, statistical code) accessible to end-users of multilevel research reports?

***Glossary terms for Characteristic 7:*** N/A

**References:**

1. Bash KL, Howell Smith MC, Trantham PS. A systematic methodological review of hierarchical linear modeling in mixed methods research. J Mix Methods Res. 2021;15:190–211.

2. Cresswell JW, Plano Clark VL. Collecting data in mixed methods research. Designing and conducting mixed methods research. 3rd ed. Thousand Oaks, CA: Sage Publications; 2018. p. 173–208.

3. Aarons GA, Green AE, Palinkas LA, Self-Brown S, Whitaker DJ, Lutzker JR, et al. Dynamic adaptation process to implement an evidence-based child maltreatment intervention. Implement Sci. 2012;7:32.

4. Headley MG, Plano Clark VL. Multilevel mixed methods research designs: advancing a refined definition. J Mix Methods Res. 2020;14:145–63.

5. Creswell JW, Creswell JD. Research design: qualitative, quantitative, and mixed methods approaches. 5th ed. Thousand Oaks, CA: Sage Publications; 2018.

6. Patton M. Qualitative research & evaluation methods. 4th ed. Thousand Oaks, CA: Sage Publications, Inc.; 2015.

7. Aguinis H, Molina-Azorín JF. Using multilevel modeling and mixed methods to make theoretical progress in microfoundations for strategy research. Strateg Organ. 2015;13:353–64.

8. Bunger AC, Navarro EI, Lewis CC. How do peers shape mental health clinicians’ attitudes toward new treatments? Adm Policy Ment Health. 2021;48:440–9.

9. Guetterman TC, Fetters MD, Creswell JW. Integrating quantitative and qualitative results in health science mixed methods research through joint displays. Ann Fam Med. 2015;13:554–61.

10. Guetterman TC, Fàbregues S, Sakakibara R. Visuals in joint displays to represent integration in mixed methods research: a methodological review. Methods Psychol. 2021;5:100080.

11. Palinkas LA, Aarons GA, Horwitz S, Chamberlain P, Hurlburt M, Landsverk J. Mixed method designs in implementation research. Adm Policy Ment Health. 2011;38:44–53.

12. Lincoln YS, Guba EG. Naturalistic inquiry. Thousand Oaks, CA: Sage; 1985.

13. Miles MB, Huberman AM, Saldaña J. Qualitative data analysis: methods sourcebook. 4th ed. Thousand Oaks, CA: Sage; 2020.

14. Morse J. Why the Qualitative Health Research (QHR) review process does not use checklists. Qual Health Res. 2021;31:819–21.

15. Tabachnick BG, Fidell LS. Using multivariate statistics. 7th ed. Pearson; 2019.

16. Snijders TAB, Bosker RJ. Multilevel analysis: an introduction to basic and advanced multilevel modeling. 2nd ed. Thousand Oaks, CA: Sage Publications Ltd.; 2011.

17. Molenberghs G, Verbeke G. Linear mixed models for longitudinal data. New York, NY: Springer New York; 2000.

18. Bryk AS, Raudenbush SW. Hierarchical linear models: applications and data analysis methods. Newbury Park, CA: Sage Publications, Inc.; 1992.

19. Hardin JW, Hilbe JM. Generalized estimating equations. Chapman and Hall/CRC; 2002.

20. Hanley JA. Statistical analysis of correlated data using generalized estimating equations: an orientation. Am J Epidemiol. 2003;157:364–75.

21. Preacher KJ, Zyphur MJ, Zhang Z. A general multilevel SEM framework for assessing multilevel mediation. Psychol Methods. 2010;15.

22. Rabe-Hesketh S, Skrondal A, Zheng X. Multilevel structural equation modeling. Handbook of Latent Variable and Related Models. Elsevier; 2007. p. 209–27.

23. Allison PD. Fixed effects regression models (Series: Quantitative Applications in the Social Sciences). Sage Publications, Inc.; 2009.

24. Cafri G, Hedeker D, Aarons GA. An introduction and integration of cross-classified, multiple membership, and dynamic group random-effects models. Psychol Methods. 2015;20:407–21.

25. Hofmann D. Centering decisions in hierarchical linear models: implications for research in organizations. J Manage. 1998;24:623–41.

26. Enders CK, Tofighi D. Centering predictor variables in cross-sectional multilevel models: A new look at an old issue. Psychol Methods. 2007;12:121–38.

27. Powell BJ, Waltz TJ, Chinman MJ, Damschroder LJ, Smith JL, Matthieu MM, et al. A refined compilation of implementation strategies: results from the Expert Recommendations for Implementing Change (ERIC) project. Implement Sci. 2015;10.

28. Williams NJ, Preacher KJ, Allison PD, Mandell DS, Marcus SC. Required sample size to detect mediation in 3-level implementation studies. Implement Sci. 2022;17:66.

29. Stanhope V, Choy-Brown M, Williams N, Marcus SC. Implementing person-centered care planning: a randomized controlled trial. Psychiatr Serv. 2021;72:641–6.

30. González-Romá V, Hernández A. Conducting and evaluating multilevel studies: recommendations, resources, and a checklist. Organ Res Methods. 2022;online.

31. Hedeker, D.H. & Gibbons, R.D. Longitudinal data analysis. Wiley; 2006.

32. McNeish D, Stapleton LM, Silverman RD. On the unnecessary ubiquity of hierarchical linear modeling. Psychol Methods. 2017;22:114–40.

33. Hubbard AE, Ahern J, Fleischer NL, Laan M Van der, Lippman SA, Jewell N, et al. To GEE or not to GEE. Epidemiol. 2010;21:467–74.
