# Supplementary material for: Eight characteristics of rigorous multilevel implementation research: a step-by-step guide
Source: Implement Sci. 2023 Oct 23;18:52. doi: 10.1186/s13012-023-01302-2 (PMC10594828; doi:10.1186/s13012-023-01302-2)
Supplement: Supplementary file 8 — Additional file 8: Characteristic 8. Ensure inferences are made at the appropriate level. [file 13012_2023_1302_MOESM8_ESM.docx]

**Additional File 8.**

***Characteristic 8:*** Ensure inferences are made at the appropriate level.

***Examples of how to spot atomistic and ecological fallacies:***

*Atomistic fallacy implementation research example: Financial incentives for implementation.* When distributed to individual providers, financial incentives may effectively increase providers’ use of an EBP with fidelity. However, that does not mean that providing financial incentives to agencies will increase overall rates of EBP use with fidelity at the agency level. This is because the meaning and implications of a financial incentive may differ for agencies versus individuals. In addition, how agencies choose to use the financial incentives may vary across organizations, with some potentially allocating the funds in ways that do not increase EBP use. Thus, an effective strategy at the individual level (e.g., increasing financial incentives) may not be effective at the agency level.

*Ecological fallacy implementation research example: Readiness to change.*

We chose readiness for change because it applies to both individuals and organizations [1,2]. An organization high in readiness for change is likely to have the structures and resources in place to support the implementation of a new practice. However, at the individual level, a provider may be high in readiness for change but lack the power to ensure that the structures and resources are in place to make the implementation successful. Thus, conclusions about the strength of the relationship between readiness for change and implementation outcomes at the organizational level should not be applied to the individual level.

***Practical considerations:*** As highlighted by Chan [3], attending to the previous seven characteristics puts researchers in a strong position to avoid erroneous interpretations of their results. For instance, by being clear about the level of the constructs (Characteristic 2), how constructs are related to each other within and across levels (Characteristic 3), and what analyses are most appropriate to test those relationships (Characteristic 7), researchers will have already thought through the multilevel issues in their data and will be more likely to interpret their analyses correctly. Implementation researchers can aid readers by being explicit in their Discussion sections about the levels of analysis, including using labels of their constructs that make clear the correct level for interpreting the results (e.g., individual-level or clinic-level or agency-level). Implementation researchers may also choose to explicitly highlight how findings at a particular level do *not* imply that similar findings would be found at a different level.

***Prompts to consider to ensure that atomistic and ecological fallacies are not present:***

□ Are our interpretations of the findings in line with how we have defined, measured, and analyzed our data?
□ Are we inferring group-level relationships based on associations between variables at the individual level? [atomistic fallacy]
□ Are we inferring individual-level relationships based on associations between variables at the group level? [ecological fallacy]
□ Are we using clear labels in our Discussion sections to ensure that readers properly understand and interpret our findings from a multilevel perspective?

***Glossary terms for Characteristic 8:*** Level of analysis, Atomistic fallacy, Ecological fallacy.

**References:**

1. Weiner BJ, Amick H, Lee S-YD. Review: Conceptualization and measurement of organizational readiness for change. Med Care Res Rev. 2008;65:379–436.

2. Rafferty AE, Jimmieson NL, Armenakis AA. Change readiness. J Manage. 2013;39:110–35.

3. Chan D. Multilevel research. In: Leong FTL, Austin JT, editors. The psychology research handbook. 2nd ed. Thousand Oaks, CA: Sage; 2006. p. 401–18.

**5 additional references that we recommend for Characteristic 8:**

Bliese, P. D., & Jex, S. M. Incorporating a mulitilevel perspective into occupational stress research: theoretical, methodological, and practical implications. J Occup Health Psychol. 2002;7:265-276.

Diez-Roux, A. V. Multilevel analysis in public health research. Annu. Rev. Public Health. 2000;21:171-192.

Diez-Roux AV. Bringing context back into epidemiology: variables and fallacies in multilevel analysis. American J public health. 1998;88:216-222.

Firebaugh G. A rule for inferring individual level relationships from aggregate data. Am Sociol Rev. 1978;43:557-572.

Robinson, W. S. Ecological correlations and the behavior of individuals. Am. Sociol. Rev. 1950;15:351–357.
