# Supplementary material for: Eight characteristics of rigorous multilevel implementation research: a step-by-step guide
Source: Implement Sci. 2023 Oct 23;18:52. doi: 10.1186/s13012-023-01302-2 (PMC10594828; doi:10.1186/s13012-023-01302-2)
Supplement: Supplementary file 9 — Additional file 9: An Integrated Example. The ASPIRE trial. [file 13012_2023_1302_MOESM9_ESM.docx]

**Additional File 9. An Integrated Example: The ASPIRE trial**

***Rationale for selecting this example***. To illustrate how the characteristics we propose might be operationalized, we present one example of their application within a mixed-methods hybrid type III effectiveness-implementation trial entitled, “Adolescent and child Suicide Prevention in Routine clinical Encounters” (ASPIRE) [1]. The ASPIRE trial offers a unified illustrative example of our proposed characteristics because it incorporates (a) multiple levels of sampling with nested observations, (b) variables (i.e., antecedents, mediators, and outcomes) that occur at different levels from each other, (c) constructs which represent shared unit characteristics that are measured through aggregation of individual responses, (d) randomization at the cluster level, and (e) both quantitative and qualitative analyses. As is the case with real-world implementation studies, it is an imperfect example and was designed to be deployable within the context of healthcare delivery settings.

***Brief overview.*** The trial focuses on improving the delivery of an evidence-based secure firearm storage program called *S.A.F.E. Firearm* in pediatric primary care practices as a universal suicide prevention strategy. It tests the comparative effectiveness of two implementation strategies and examines the mechanisms through which those strategies relate to improved implementation. In the trial, primary care practices are randomly assigned to either a less costly, more scalable strategy integrated into the electronic health record (called ‘*Nudge*’) or a more costly, more intensive external facilitation strategy plus the electronic health record strategy (called ‘*Nudge+*’). The primary outcome is reach of *S.A.F.E. Firearm,* measured at the level of patient-caregiver dyads using a binary indicator of exposure versus non-exposure. This outcome is measured based on information in the electronic medical record.

Mixed methods, including quantitative multilevel analyses and qualitative interviews, will be used to test the strategies’ hypothesized mechanisms and to uncover additional mechanisms through which the strategies’ influence reach. The focal quantitative mediator is practice-level adaptive reserve, measured by clinician-reported surveys, and defined as the collective ability of teams within each practice to make and sustain change (at the practice level) through problem-solving and tailoring of the implementation process to the local practice.

***Characteristic 1: Map and operationalize the specific multilevel context for defined populations and settings.***

1. Create and include a list or map of contextual levels most salient to the research question and population under study.

In partnership with system and primary care partners, the research team identified the levels of the implementation context that are relevant to the study’s main hypotheses. These levels and their rationale are shown in Table A1.

**Table A1. Salient levels of implementation context in the ASPIRE trial.**

| **Level Name** | **Definition** | **Rationale for Inclusion** |
| --- | --- | --- |
| Health system | Following the definition of proposed by the Agency for Healthcare Research and Quality in 2016, health systems are defined as: “an organization that includes at least one hospital and at least one group of physicians that provides comprehensive care (including primary and specialty care) who are connected with each other and with the hospital through common ownership or joint management.” | One of the focal populations for the study is primary care practices which are typically embedded within/ members of health systems. Health systems are an important level in the implementation context because they control funding and policy that influence care provided within primary care practices |
| Primary care practice | Primary care practices are defined as privately-held, for-profit or non-profit, outpatient clinics, which typically represent standalone buildings, which house a set of providers who deliver care to children and youth. | In the USA, where this study is underway, most pediatric primary care is delivered in primary care practices; therefore, this is an important population of practice settings within which to understand how to improve the implementation of pediatric suicide prevention interventions. The implementation strategies are also assigned at the level of primary care practices so this is an essential level. In addition, the mediator is conceptualized as a characteristic of primary care practices. |
| Clinicians | Clinicians are defined as licensed care team members qualified to prescribe medication and refer to specialists; these included physicians, nurse practitioners, and physician assistants. | Within primary care practices, care is delivered to patients by clinicians. The *S.A.F.E Firearm* intervention is designed to be delivered by clinicians to patients during routine care. |
| Youth-caregiver dyad | Youth patients (ages 5-17 years), accompanied by their parents or legal guardians | Youth are the target of *S.A.F.E. Firearm*; consequently, it is important to examine implementation/clinical outcomes for this population. The primary outcome is **reach** of *S.A.F.E. Firearm,* measured at the level of patient-caregiver dyads using a binary indicator of exposure versus non-exposure based on information in the electronic medical record. |

***Characteristic 2:*** ***Define and state the level of each construct under study***

1. For each construct, define its substantive meaning and the level at which it resides/ population unit with which it is associated.
2. For each construct, provide an explanation or ‘mini theory’ that explains why the construct is assigned to its specific level/ population unit.

Table A2 lists the constructs under study in the ASPIRE trial and their associated level/population unit.

**Table A2. Construct definitions and levels in the ASPIRE trial.**

| **Construct** | **Substantive Definition** | **Level/ Population Unit** | **Theoretical rationale for level** |
| --- | --- | --- | --- |
| Implementation strategy condition | This is the study’s primary antecedent variable. It is defined and operationalized as covariate constrained random assignment to *Nudge* (electronic health record intervention) vs. *Nudge+* (electronic health record intervention + external facilitation). | Primary care practice | This variable occurs at the level of primary care practices because (a) randomization occurred at the level of primary care practices, and (b) the implementation strategies (modification of electronic health record and facilitation) target entire primary care practices for change. |
| Practice adaptive reserve | This is the study’s primary (only) quantitative mediator for the analysis of mechanisms. It is defined as the collective capacity within the practice to make and sustain change in support of implementing newly introduced clinical interventions. Measured using the Practice Adaptive Reserve Scale [2], a staff-reported measure that assesses relationship infrastructure, facilitative leadership, sense-making, teamwork, work environment, and culture of learning. | Primary care practice | This variable occurs at the level of primary care practices because theory on adaptive reserve indicates it is a collective (i.e., shared) characteristic of practices, not of individual clinicians within the practices. |
| Reach of *S.A.F.E. Firearm* | This is the study’s primary implementation outcome. It is defined and operationalized as youth-caregiver dyad’s receipt or nonreceipt of *S.A.F.E. Firearm* as documented in the electronic health record. | Youth-caregiver dyad | This variable occurs at the youth-caregiver dyad because *S.A.F.E. Firearm* is designed to be delivered to individual youth and their caregivers during primary care clinical encounters. |

***Characteristic 3:*** ***Describe how constructs relate to each other within and across levels.***

1. Include a figure or narrative that describes the study’s theoretical model, including the level of each construct and the hypothesized relationships between constructs.
2. When hypothesized relationships cross levels, researchers should describe the processes through which higher-level antecedents influence lower-level consequents (i.e., top-down processes) or how lower-level antecedents shape higher-level consequents (i.e., bottom-up processes).
3. Clarify each construct’s location in the study theoretical model relative to other constructs (e.g., is it an antecedent, mediator, consequent, primary or secondary endpoint, etc.).

Figure A1 presents ASPIRE’s theoretical model.^[[1]](#footnote-1)^ In the model, assignment to implementation strategy is the primary antecedent. NUDGE is hypothesized to increase the reach of *S.A.F.E. Firearm* by providing a reminder to clinicians within the electronic health record; the reminder is designed to overcome limitations in individual clinician resources (e.g., attention, time) which may prevent their delivery of *S.A.F.E. Firearm* to youth-caregiver dyads during clinical encounters. Behavioral economics theory explains how context and limited resources (e.g., time, attention) influence provider’s practice behaviors through the use of cognitive heuristics or shortcuts in decision-making [3,4]. It proposes that cognitive heuristics can be leveraged to influence behavior through choice architecture which involves structuring the environment to facilitate a target choice. The *Nudge* strategy applies choice architecture to the electronic health record to increase the likelihood that clinicians will deliver *S.A.F.E Firearm* to youth and their caregivers during clinical encounters.

**Figure A1. ASPIRE Theoretical Model**


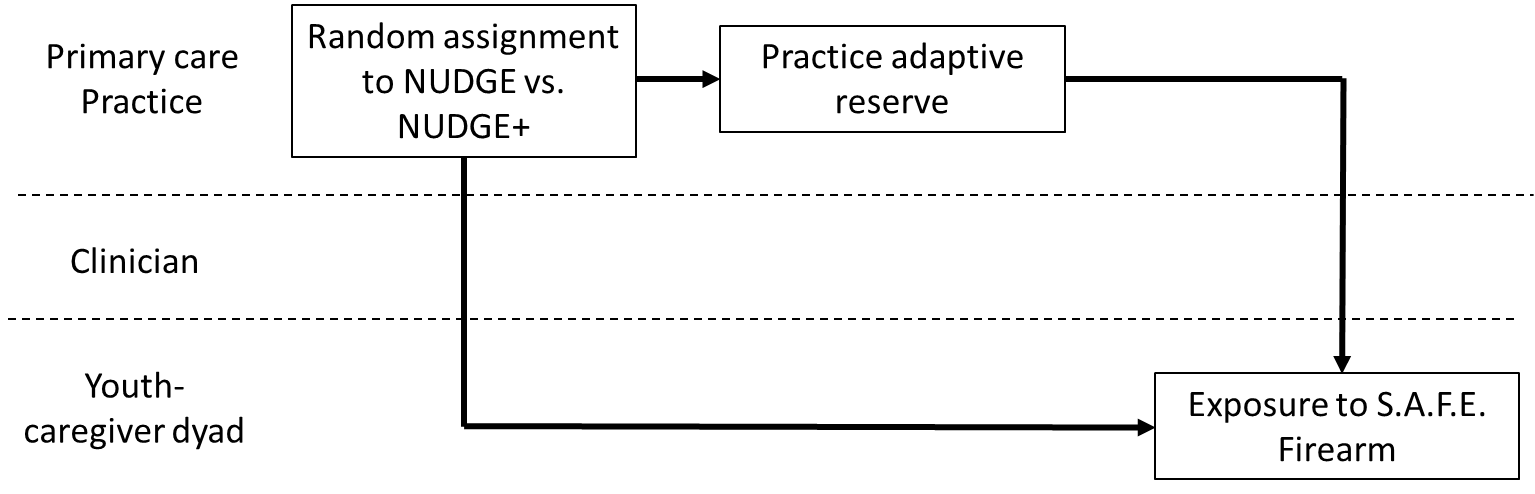


*Nudge*+ also incorporates a reminder in the electronic health record but adds to it a facilitation strategy. Theories of practice facilitation propose that implementation can be improved through partnerships between trained facilitators and local providers who work together to identify and address site-specific implementation barriers as well as organizational adaptive reservice (i.e., the capacity for improvement and sustainment of the desired practice) [5,6]. *Nudge*+ draws on these theories to argue that the use of facilitation will increase practice adaptive reserve, and in turn, the likelihood that youth and their caregivers receive *S.A.F.E. Firearm* during clinical encounters.

Adaptive reserve is a mediator of the relationship between implementation condition and reach. Adaptive reserve is expected to increase the extent to which youth-caregiver dyads receive *S.A.F.E. Firearm* during clinical encounters, and therefore to increase the practice-level proportions of this outcome, by enabling clinicians and practice leaders to identify and address site-specific implementation barriers to *S.A.F.E. Firearm*.

The primary implementation outcome is reach of *S.A.F.E. Firearm*.

In addition to the hypothesized relationships shown in Figure A1, qualitative interviews with purposefully sampled clinicians and with practice leaders will provide additional information about mechanisms through which the two implementation strategies may have influenced *S.A.F.E. Firearm* implementation. The goal of these interviews is to identify additional mechanisms (including potentially bottom-up mechanisms) through which the implementation strategies may have influenced the reach of *S.A.F.E. Firearm*. The interview guide will be developed using the Consolidated Framework for Implementation Research [7] and the sampling strategy will purposefully target clinicians who achieved high and low reach as well as those who report firearm ownership.

***Characteristic 4:*** ***Specify the temporal scope of each phenomenon at each relevant level.***

Provide a detailed explanation of the expected temporal dynamics within the study at each level, using visual aids as needed, to include the following:

1. When investigators expect to observe change in each relevant outcome at each relevant level (e.g., of system- or organization-level implementation strategies),
2. How frequently and when constructs will be measured to capture these changes,
3. How changes in outcomes at different levels align with each other in the research design,
4. The theoretical rationale for these choices.

One of the implementation strategies, *Nudge*, requires modification of the electronic health record. Once the modification is operationally defined and installed, it will instantaneously take effect and should begin influencing the implementation outcome immediately. The other implementation strategy, *Nudge*+, involves modification to the electronic health record plus 12 months of facilitation to increase practices’ adaptive reserve. Theory and prior empirical research are silent on the issue of how long it will take for practices to develop adaptive reserve. In the ASPIRE trial, the mediation analysis will be structured in such a way that the gradual improvement in adaptive reserve from baseline to follow-up is analyzed as a mediator of the difference in youth-caregiver exposure to S.A.F.E. Firearm during the implementation and sustainment phases. This timing is reflected in the study design by measuring the reach outcome for an entire 12 months during the active implementation phase (which begins after the electronic health record is modified) and then again for 12 months during a ‘sustainment’ phase.

Qualitative interviews with leaders and clinicians may provide additional information about the timing of changes that influenced *S.A.F.E. Firearm* reach.

***Characteristic 5:*** ***Align measurement choices and construction of analytic variables with the levels of theories selected (and hypotheses generated, if applicable).***

Align the levels of theory and measurement. For unit-level constructs, determine whether the construct is a global, shared, or configural property of the unit and use this to align measures with theory. For shared constructs, address the following:

1. Include a specific referent that indicates who and/or what is being rated,
2. Effectively communicate these referents to participants in measurement instruments,
3. Ensure respondents who are asked to report on shared constructs can report on them and that they are the appropriate persons to ask,
4. Provide evidence that individuals within a unit reflect (and can report on) a shared phenomenon or experience,
5. When shared constructs are measured quantitatively using individual responses, aggregate the individual responses into unit-level scores shared constructs.

In the ASPIRE trial, assignment to implementation condition is a global construct because entire primary care practices are assigned to condition as units. Adaptive reserve is a shared construct that occurs at the level of primary care practices. It is a shared construct because it represents the *collective* capacity within the practice to make and sustain change in support of implementing newly introduced clinical interventions. As such, it is analyzed at the level of primary care practices. The measure of practice adaptive reserve is administered to individual clinicians who make individual ratings. These individual ratings will be aggregated (averaged) to the practice-level to represent the shared construct.

To ensure individual clinician responses represent ratings of a shared construct, items on the practice adaptive reserve measure refer specifically to the primary care practice within which clinical staff work. That is, the referent is the respondent’s primary care practice. Some items on the practice adaptive reserve scale assess practice leadership and these are worded so that they refer specifically to leaders and leader behaviors within the respondent’s primary care practice. Examples of items include:

*“People in our practice actively seek new ways to improve how we do things.”*

*“Leadership in this practice creates an environment where things can be accomplished.”*

The setup of workflows within primary care practices involve interaction between staff and ensure that all staff can reasonably rate the items they are asked about.

To assess the validity of the shared construct of practice adaptive reserve, the research team will calculate measures of inter-rater agreement within each primary care practice in order to show that staff within a practice agree with each other on their perceptions. The index of inter-rater agreement will be rwg(j) or awg(j). In addition, an intraclass correlation coefficient (ICC[1]) or analysis of variance (ANOVA) will be calculated, with practice as the factor, to assess the extent of variation across practices in clinician perceptions of practice adaptive reserve. Use of these procedures will support the construct validity of the aggregated, practice-level adaptive reserve variable in two ways. First, evidence of within-practice inter-rater agreement confirms that clinicians within each practice agree on their perceptions of adaptive reserve. This provides confirmation that adaptive reserve is a shared, practice-level construct that is similarly experienced by (and can therefore influence) all members of the practice. Second, measures of ICC(1) and/or the ANOVA, confirms there is significant variation in the mean level of adaptive reserve across practices. This is important because it demonstrates that adaptive reserve varies across practices and is therefore not something universally agreed upon by all staff across all practices. Variation in adaptive reserve across practices is also necessary in order for the variable to serve as a mediator of the implementation strategy’s effects on *S.A.F.E. Firearm* reach.

In analytic models, the adaptive reserve variable will be entered at the level of primary care practices. Models will be specified such that assignment to implementation condition influences practice-level means of adaptive reserve, which in turn influence youth-caregiver dyad exposure to *S.A.F.E. Firearm*.

Qualitative interviews with practice leaders and clinicians may provide additional information about the validity of aggregate constructs as well as other important reference leaders or groups that may be salient for understanding *S.A.F.E. Firearm* implementation.

***Characteristic 6:*** ***Use a sampling strategy consistent with the selected theories or research objectives and sufficiently large and variable to examine relationships at requisite levels.***

Design and justify a multilevel sampling plan, ensuring there is:

1. A large enough sample at each level to rigorously test hypotheses or make theoretical inferences,
2. Adequate *variability* within the sample *at each level* to rigorously test hypotheses or make theoretical inferences, and
3. Adequate *representativeness* of the achieved sample *at each level* (for quantitative).

When reporting study findings include:

1. The distribution and range of within-unit sample sizes,
2. The distribution and range of within-unit response rates,
3. A (statistical, if possible) comparison of the characteristics of unit members who responded versus those who did not respond,
4. The theoretical or empirical rationale for exclusion of units (as applicable).

The ASPIRE trial sampling plan includes three levels: primary care practices, clinicians, and youth-caregiver dyads. Each level is essential to the hypotheses being tested, inextricably linked within the health system, and worth the data collection burden.

The study incorporates 30 practices (15 per condition), a sample shown through statistical power analyses to be sufficient to adequately power the primary hypothesis test. Historical administrative data and information from pilot work indicates that the number of clinicians in each practice and the number of child-caregiver dyads served per clinician during the study period is sufficiently large, given the eligibility criteria, to ensure variation across practices in the primary outcomes and stable estimates of the primary parameters. The introduction of the facilitation strategy *Nudge*+ to half the practices is expected to generate sufficient variability in practice adaptive reserve and reach of *S.A.F.E. Firearm* across the two conditions to permit a test of the primary hypothesis.

The representativeness of the sample at each level (e.g., clinicians per practice, youth-caregiver dyads per clinicians) cannot be assessed until after data collection is complete. However, procedures are in place to include all eligible clinicians and youth-caregiver dyads in each practice. Procedures are also in place to monitor the representativeness and comprehensiveness of clinician and youth-caregiver dyad recruitment; these will be a focal point of monitoring study operations and quality control. Reporting of the items recommended above will occur when primary study results are presented.

Plans are in place for qualitative interview data to stratify sampling by relevant clinician and practice characteristics as well as to ensure purposive sampling continues until thematic saturation is reached (in the case of clinicians) or until all leaders are sampled (in the case of leaders).

***Characteristic 7:*** ***Align analytic approaches with the chosen theories (and hypotheses, if applicable), ensuring that they account for measurement dependencies and nested data structures.***

1. Directly acknowledge dependencies (i.e., correlated observations/ nesting) within the proposed study design, articulate what statistical method has been selected to account for those dependencies, and provide a rationale for the choice of model with reference to specific characteristics of the data and strengths of the selected model,
2. Ensure that variables enter statistical models at the level warranted, and scrutinize choices related to centering, standardization, and calculation of effect sizes to confirm they reflect the study’s multilevel design; for randomized studies, the variable representing randomization to condition (i.e., exposure) should enter the statistical model at the level at which randomization occurs,
3. Be transparent and thorough in reporting details of the analytic approach,
4. Consider developing and sharing crosswalks that specify research questions and justify the use of data collection tools and their accompanying analytic techniques, defining their multilevel purpose and (anticipated) contributions, including “explicit connections” or “intentional redundancies” among quantitative and qualitative approaches.

In the ASPIRE trial, observations of the primary outcome, *S.A.F.E. Firearm* reach occur at the level of youth-caregiver dyads based on clinician documentation in the electronic health record. Youth-caregiver dyads complete once-per-year well-child visits and their exposure to *S.A.F.E. Firearm* will be assessed during these visits. Observations of reach for each youth-caregiver dyad will be nested within clinicians (who serve multiple youth-caregiver dyads), and clinicians will be nested within practices. This nesting is likely to induce dependence (correlations) among observations of reach which will need to be addressed in the analyses. Observations may be dependent (correlated) within the two health systems; however, this is unlikely based on prior research. The study therefore has three levels of nesting to account for in the analyses: primary care practice (the level at which randomization occurs), clinician, and youth-caregiver dyad.

Dependent observations within clinicians and practices will be accounted for using generalized estimating equations which are ideal for addressing clustered observations when the outcome is binary because they yield marginal effect interpretations and are robust to misspecification of the correlation structure [8,9]. Preliminary analyses will assess the extent of clustering at the health system level and, if necessary, a fixed effect (indicator variable) for health system will be added to the models to account for health system effects on the outcome. In all analyses, the variable ‘assignment to implementation condition’ will enter models at the practice level in accordance with the level of randomization. The primary outcome, youth-caregiver dyad exposure to *S.A.F.E. Firearm*, will be analyzed at that level.

The mediation analysis incorporates a practice-level independent variable (NUDGE v. NUDGE+), a practice-level mediator (adaptive reserve), and a youth-caregiver dyad-level outcome (reach); thus, it represents a 3-3-1 model, where the numbers in the sequence represent the level of the X (independent variable), M (mediator), and Y (outcome) variables in the design. Because the X and M variables are at the same level and are linked to the outcome through a GEE model, the model is appropriately analyzing practice-level variance in all three variables and thus the model is properly specified. The indirect effect can be estimated using the product of coefficients approach or through causal effects formulas that address clustered observations.

***Characteristic 8: Ensure inferences are made at the appropriate level.***

Carefully craft and check language within research reports and presentations to ensure atomistic and ecological fallacies are not present.

Given the design and statistical model employed for the primary outcomes, inferences from the ASPIRE trial will focus on the marginal probability of youth-caregiver dyad receipt of *S.A.F.E. Firearm* by condition. An alternative conceptualization of reach in the ASPIRE trial would focus on the practice-level *ratio* of exposed youth-caregiver dyads relative to the number of eligible youth-caregiver dyads (e.g., 60% exposed vs. 50% exposed); however, this is not the focus of the ASPIRE trial.

**References**

1. Beidas RS, Ahmedani BK, Linn KA, Marcus SC, Johnson C, Maye M, et al. Study protocol for a type III hybrid effectiveness-implementation trial of strategies to implement firearm safety promotion as a universal suicide prevention strategy in pediatric primary care. Implement Sci. 2021;16:89.

2. Nutting PA, Crabtree BF, Stewart EE, Miller WL, Palmer RF, Stange KC, et al. Effect of facilitation on practice outcomes in the National Demonstration Project model of the patient-centered medical home. Ann Fam Med. 2010;8:S33-44.

3. Tversky A, Kahneman D. The framing of decisions and the psychology of choice. Science. 1981;211:453–8.

4. Patel MS, Day SC, Halpern SD, Hanson CW, Martinez JR, Honeywell S, et al. Generic medication prescription rates after health system–wide redesign of default options within the electronic health record. JAMA Intern Med. 2016;176:847-848.

5. Baskerville NB, Liddy C, Hogg W. Systematic review and meta-analysis of practice facilitation within primary care settings. Ann Fam Med. 2012;10:63-74.

6. The practice facilitation handbook: training modules for new facilitators and their trainers. [Internet]. 2013. Available from: https://www.ahrq.gov/sites/default/files/publications/files/practicefacilitationhandbook.pdf.

7. Damschroder LJ, Aron DC, Keith RE, Kirsh SR, Alexander JA, Lowery JC. Fostering implementation of health services research findings into practice: a consolidated framework for advancing implementation science. Implement Sci. 2009;4:50.

8. Hubbard AE, Ahern J, Fleischer NL, Laan M Van der, Lippman SA, Jewell N, et al. To GEE or not to GEE. Epidemiol. 2010;21:467–74.

9. Liang KY, Zegler SL. Longitudinal data analysis using generalized linear models. Biometrika. 1986;73:13–22.

1. We note that the ASPIRE trial also includes moderation hypotheses that examine under what conditions the implementation strategies are most effective; however, we do not discuss those here for the sake of space. [↑](#footnote-ref-1)
